# Human tripartite motif protein 52 is required for cell context-dependent proliferation

**DOI:** 10.18632/oncotarget.24422

**Published:** 2018-02-05

**Authors:** Stefan Benke, Benedikt Agerer, Lisa Haas, Martin Stöger, Alexander Lercher, Lisa Gabler, Izabella Kiss, Sara Scinicariello, Walter Berger, Andreas Bergthaler, Anna C. Obenauf, Gijs A. Versteeg

**Affiliations:** ^1^ Department of Microbiology, Immunobiology, and Genetics, Max F. Perutz Laboratories, University of Vienna, Vienna Biocenter, Vienna 1030, Austria; ^2^ Research Institute of Molecular Pathology, Vienna Biocenter, Vienna 1030, Austria; ^3^ CeMM Research Center for Molecular Medicine of the Austrian Academy of Sciences, Vienna 1090, Austria; ^4^ Institute of Cancer Research and Comprehensive Cancer Center, Department of Medicine I, Medical University of Vienna, Vienna A-1090, Austria

**Keywords:** tripartite motif protein (TRIM), glioblastoma, proliferation, p53, glucose metabolism

## Abstract

Tripartite motif (TRIM) proteins have been shown to play important roles in cancer development and progression by modulating cell proliferation or resistance from cell death during non-homeostatic stress conditions found in tumor micro-environments. In this study, we set out to investigate the importance for cellular fitness of the virtually uncharacterized family member TRIM52.

The human *TRIM52* gene has arisen recently in evolution, making it unlikely that TRIM52 is required for basic cellular functions in normal cells. However, a recent genome-wide ablation screening study has suggested that TRIM52 may be essential for optimal proliferation or survival in certain genetic cancer backgrounds. Identifying genes which fit this concept of genetic context-dependent fitness in cancer cells is of interest as they are promising targets for tumor-specific therapy.

We report here that *TRIM52* ablation significantly diminished the proliferation of specific glioblastoma cell lines in cell culture and mouse xenografts by compromising their cell cycle progression in a p53-dependent manner. Together, our findings point to a non-redundant TRIM52 function that is required for optimal proliferation.

## INTRODUCTION

Mammals encode approximately 600 proteins harboring a Really Interesting New Gene (RING) zinc finger domain [1]. Many of these proteins are ubiquitin E3 ligases, which mediate the covalent attachment of the post-translational modifier-protein ubiquitin to lysine residues in target proteins, thereby altering their localization, activity, stability, or function. Conceptually, RING proteins facilitate these reactions by binding both a ubiquitin-loaded E2 conjugase enzyme through its RING domain, as well as a substrate through another domain, and bringing them in close proximity for ubiquitin transfer onto the substrate to occur [1].

Two proportionally sized loops protruding from the RING domain zinc-finger are essential for binding of E2 enzymes, and hence E3 activity of RING proteins [1]. Some RING proteins have disproportionally sized loops and are therefore often unable to bind E2 enzymes, and as such unable to function as direct E3 ligases [2]. The two most extreme RING loops are found in the two closely related paralogs Tripartite Motif protein 41 (TRIM41) and TRIM52 [3]. These RING proteins are extreme in i) the size of their RING domain -especially loop 2 of 139 aa in TRIM52 compared to an average of 13 aa in most other RING proteins-, and ii) the amino acid composition of loop 2: ∼35% aspartate and glutamate [3].

TRIM proteins have been implicated in regulation of many different cellular processes including cancer cell proliferation or resistance from cell death [4]. Phylogenetic analysis has indicated that *TRIM52* has arisen in eutherian mammals through partial gene duplication of just the RING and Bbox domains of *TRIM41,* which contains all conserved domains of most other typical TRIM proteins: RING-Bbox-Coiled Coil-B30.2 [3]. Subsequently, many mammals have lost or pseudogenized the *TRIM52* gene, whereas it has been maintained in primates and Old-World monkeys [3]. Together, these findings indicate that TRIM52 is dispensable in many mammals for development and essential homeostatic processes. Yet, primates and Old World monkeys have maintained a protein-coding *TRIM52* gene, suggesting that it plays a biological role in these species.

Various residues in TRIM52 have been under positive selection pressure, which has previously prompted the speculation that it may be involved in anti-viral defense. However, unlike some other TRIM family members, TRIM52 did not confer any resistance against lenti- and retroviral infection [4].

Thus far, the biological functions of TRIM41 and TRIM52 have remained relatively poorly investigated. TRIM41 has been reported to target various Protein Kinase C (PKC) isoforms for degradation in a ubiquitin- and proteasome-dependent manner [5, 6], yet the effect of this regulation on cell function remains unclear. In contrast, TRIM52 function has only been studied in over-expression experiments, which have suggested i) a putative antiviral function by targeting the NS2A protein of Japanese encephalitis virus for proteasomal degradation [7], and ii) a putative cellular function in activation of the pro-inflammatory NFκB response [8].

However, endogenous TRIM52 protein expression, as well as its importance for human cell homeostasis under conditions of *TRIM52* ablation had not been previously investigated. The fact that the human *TRIM52* gene has arisen recently in evolution by gene duplication of *TRIM41*, and has been lost or pseudogenized in various other mammalian species suggested it to be unlikely that TRIM52 would be required for basic cellular functions. Yet, in contrast, recent analysis of several genome-wide ablation screening studies has suggested that TRIM52 may be essential for optimal proliferation or survival of some cancer cell lines [9, 10], but not others [9, 11]. Together, these data suggest that proper expression of the *TRIM52* gene could be required for efficient proliferation or viability only in certain genetic cancer cell backgrounds. Identifying genes which fit this concept of genetic context-dependent fitness in cancer cells is of particular interest as they are promising targets for tumor-specific therapy [12]. In the current study, we tested the specific hypothesis that TRIM52 is important for cancer cell fitness in a context-specific manner.

We report here that *TRIM52* mRNA is expressed to moderate levels in all tested human cell lines. *TRIM52* ablation by inducible shRNAs significantly diminished the cell numbers of two glioblastoma cell lines by compromising their cell cycle progression in a p53-dependent manner. Together, our findings point to a non-redundant TRIM52 function that is required for optimal proliferation in certain cancer cell lines.

## RESULTS

### TRIM52 protein is lowly expressed in divergent cancer cell lines

Requirement for cellular functions of the endogenous TRIM52 protein had remained completely uncharacterized. Hence, in this study we set out to determine its importance for cellular fitness. RT-qPCR analysis had indicated that *TRIM52* mRNA is expressed to similar, moderate levels in various commonly used human cancer cell lines (approximately eight RT-qPCR cycles above the detection limit; data not shown), suggesting it was feasible to expect detectable amounts of TRIM52 protein. To investigate TRIM52 expression and the effect of its ablation, U87MG glioblastoma cells were stably transduced with doxycycline (dox)-inducible lentiviral constructs encoding either non-targeting (NT) or *TRIM52*-targeting (T52) shRNAs in the artificial 3′-UTR of GFP [13] (Supplementary Figure 1A). *TRIM52* mRNA expression was consistently decreased by ≥90% by two independent *TRIM52*-targeting shRNAs upon dox-treatment, but not two independent non-targeting controls (Supplementary Figure 1B). Moreover, the knockdown was specific for *TRIM52*, as the mRNA level of its closest paralog -*TRIM41*-, and the levels of two lncRNAs encoded in adjacent loci (*TRIM52-AS1* and *CTC338M12.4*) remained unchanged under these conditions (Supplementary Figure 1C).

**Figure 1:**
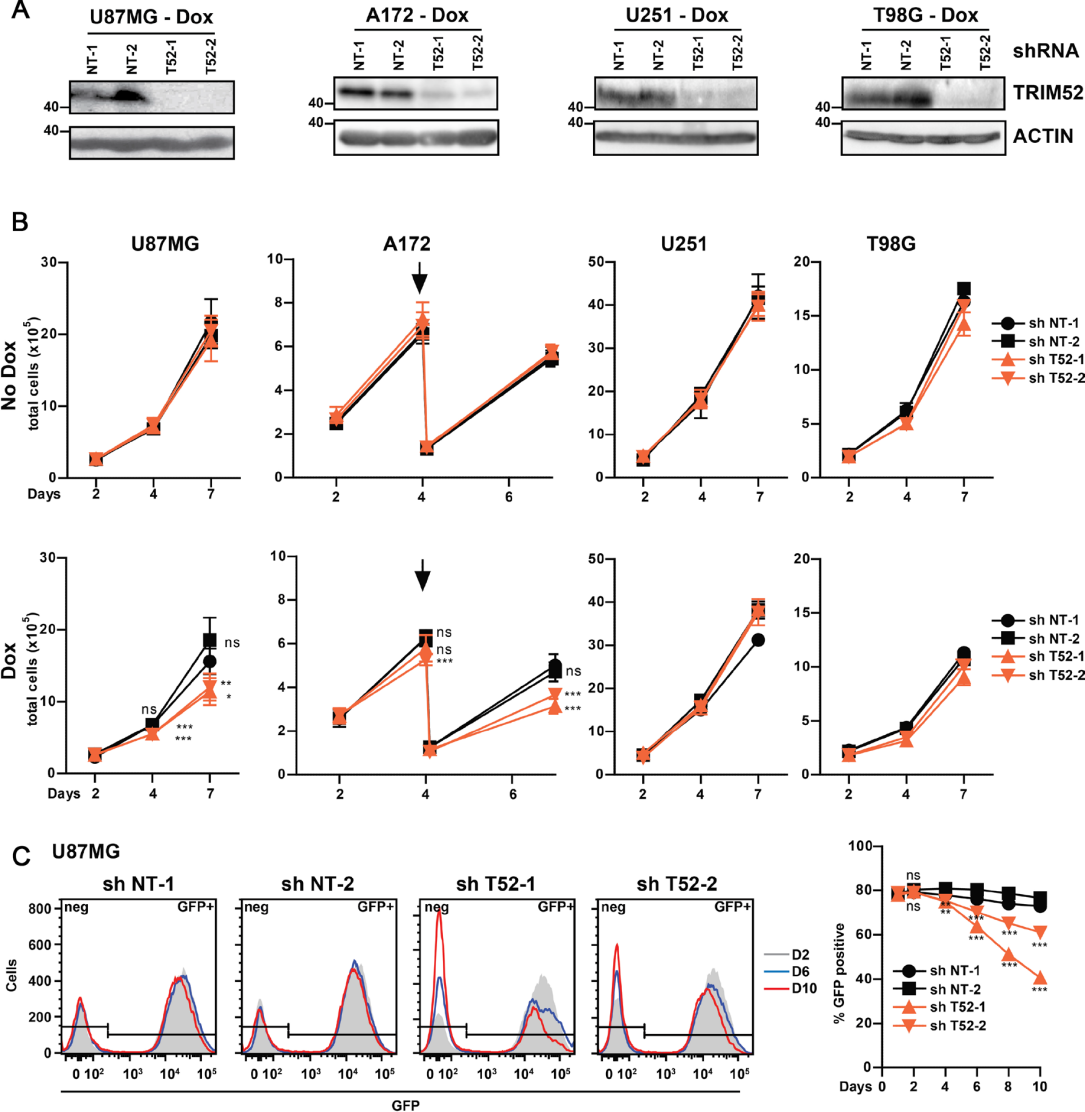
*TRIM52* ablation reduces cellular fitness in a subset of glioblastoma cell lines U87MG, A172, U251 or T98G glioblastoma cells stably transduced with dox-inducible shRNA vectors were seeded at a density of 1 × 10^5^ cells/well, and treated with dox. (**A**) TRIM52 protein expression was analyzed by western blot. (**B**) Total cells were counted 2, 4 and 7 days after seeding. A172 cells reached confluency at day 4, and were thus passaged once at a fixed ratio (1:5, indicated by arrow). Pooled data from two independent experiments (mean ± SD, *n* = 5–6; student’s *t*-test was performed compared to NT-1 control: ^*^p > 0.05; ^**^p > 0.01; ^***^p > 0.001). (**C**) U87MG cells stably transduced with dox-inducible shRNA vectors were mixed with WT cells (80% shRNA cell lines, 20% WT cells), and passaged every two days at a fixed ratio of 1:2 in the presence of dox. GFP fluorescence intensity (left panel) and percentage GFP positive cells (right panel) were measured by flow cytometry. Representative of at least three independent experiments. Data represent mean ± SD, *n* = 3; student’s *t*-test were calculated compared to non-targeting shRNAs: ^*^p > 0.05; ^**^p > 0.01; ^***^p > 0.001).

Subsequent analysis of TRIM52 protein by Western blot (WB) using a mAb showed that the ∼50 kDa product detected in cells expressing non-targeting shRNAs, was reduced to nearly undetectable levels upon specific *TRIM52*-knockdown (Figure 1A). Together, these data indicate that TRIM52 protein is specifically detected using this mAb.

### *TRIM52* ablation reduces cancer cell fitness in a cell context-dependent manner

To investigate the requirement of TRIM52 for optimal cell growth and survival, we first set out to investigate whether *TRIM52* reduction would impose a change in cellular fitness. To this end, a panel of eight human cancer cell lines of different genetic make-up and tissue origin (Supplementary Table 1) was stably transduced with dox-inducible lentiviral shRNA constructs as described above. This setup enabled maintaining stable cell lines without the risk of out-selecting cells with decreased cellular fitness.

shRNA expression was dox-induced in polyclonal cell pools for each shRNA, and directly compared to their non-induced counterparts. Subsequently, total cell numbers were counted at increasing days post-induction with dox. *TRIM52* knockdown significantly reduced the cell numbers of U87MG and A172 glioblastoma cell lines, but not the other two glioblastoma lines tested (Figure 1B), even though TRIM52 protein expression was equally reduced in all four glioblastoma cell lines (Figure 1A), and efficient knockdown was maintained throughout all days of the experiment (Supplementary Figure 1D). *TRIM52* targeting in all the other tested cell lines did also not significantly reduce total cell numbers (Supplementary Figure 2A). Together, these data show that *TRIM52* knockdown reduces growth fitness in some cancer cell lines, and thus indicate that TRIM52 expression provides a fitness advantage in a cell-context dependent manner. Moreover, the observed cell fitness phenotype was not fully penetrant for any particular cancer- or tissue-type, suggesting that rather the cell-specific genetic make-up determines whether *TRIM52* targeting confers a decrease in fitness.

Subsequently, a complementary direct competition assay was used to compare growth fitness in one of the cell lines sensitive to *TRIM52* knockdown (U87MG; Figure 1C), and in one insensitive (HeLa; Supplementary Figure 2B–2C). In this assay, non-transduced wild-type (WT) cells were mixed in a 20%:80% ratio with cells harboring inducible *TRIM52*-targeting or non-targeting shRNAs. Subsequently, these cells were treated with dox, which induces both shRNA and GFP expression, allowing us to distinguish them from WT cells. The percentage of GFP-positive cells was followed by flow cytometry during cell growth as a measure for competitive fitness. shRNA-expressing cells with reduced fitness compared to WT cells are predicted to be depleted from the culture, presenting as a reduction in the percentage of GFP-positive cells. Since *TRIM52*-targeted and WT cells were co-cultured in the same dish in this assay, any differences in proliferation are cell-autonomous and cannot stem from differences in factors released into the cell culture medium.

The percentage of U87MG cells expressing *TRIM52* shRNAs was significantly reduced in the course of ten days of mixed culture, whereas cells expressing non-targeting shRNAs were not (Figure 1C). Likewise, the percentage of HeLa cells expressing *TRIM52*-targeting constructs remained unchanged (Supplementary Figure 2B–2C), in line with the results from the cell counting experiments (Supplementary Figure 2A). Moreover, the GFP mean fluorescence intensity in *TRIM52*-targeting shRNA expressing U87MG cells moderately decreased over time (Figure 1C), suggesting that preferentially cells expressing the highest shRNA levels were out-competed. These results are in agreement with the previous cell counting assays (Figure 1B), and show that *TRIM52* depletion is detrimental for growth fitness in a cell-autonomous and cell context-dependent manner.

Subsequent analysis of protein lysates of several patient-derived glioblastoma lines, and human stem cell-derived cerebral organoids, demonstrated that TRIM52 protein was expressed in ten out of thirteen analyzed samples, albeit at varying relative levels (Supplementary Figure 2D). Expression of TRIM52 was relatively high in U87MG and A172 cells, whereas we could not detect TRIM52 protein in lysates from three patient-derived GBM lines (Supplementary Figure 2D). From these data, we conclude that TRIM52 protein is widely expressed and could contribute to proliferation in GBM cells. However, the fact that *TRIM52* knockdown affected A172 cells but not T98G cells (Figure 1B), even though TRIM52 protein levels in these cell lines are comparable (Supplementary Figure 2D), indicates that expression levels *per se* do not determine the importance of TRIM52 for optimal cell fitness.

### *TRIM52* ablation reduces tumor growth in a mouse xenograft model

Next, we investigated whether TRIM52 is required for optimal tumor growth in a U87MG xenograft mouse model. To allow *in vivo* bioluminescence imaging (BLI) as a measure for tumor size, a vector constitutively expressing firefly luciferase (Fluc) was stably integrated in U87MG cells with dox-inducible non-targeting, or *TRIM52*-targeting shRNAs.

**Figure 2:**
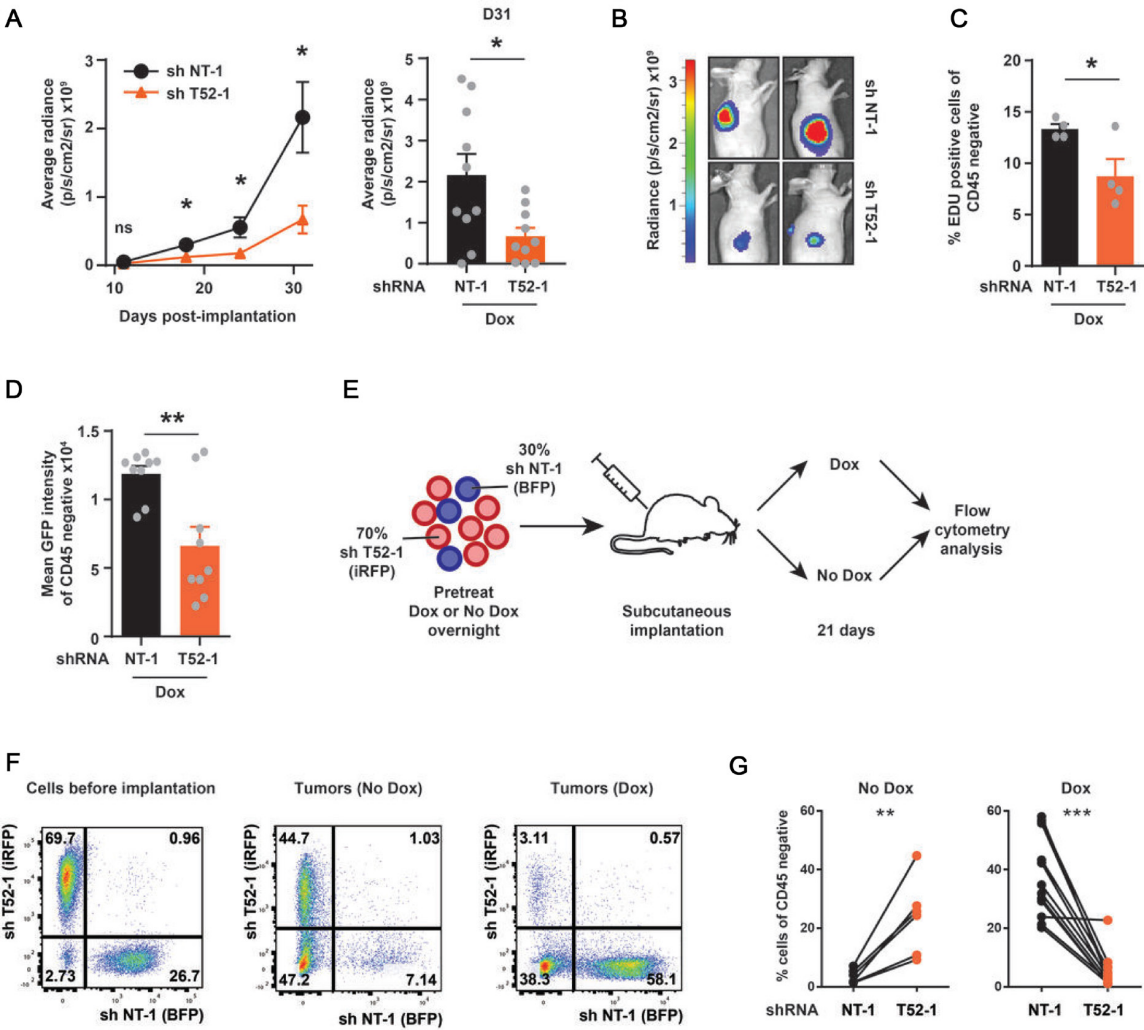
*TRIM52* ablation reduces cancer cell proliferation and tumor formation in a U87MG xenograft model *in vivo* U87MG cells stably harboring inducible non-targeting or *TRIM52*- targeting shRNA constructs were transduced with constructs encoding firefly luciferase (Fluc). Cells were pretreated with dox for 24 h and subcutaneously injected in the flanks of athymic nude mice. Mice were fed dox via drinking water for the duration of the experiment. (**A**) Bioluminescence imaging (BLI) was performed at indicated time points for all mice, and average radiance measured for each tumor. (Plotted are mean ± SEM. ^*^p < 0.05, two-tailed Mann-Whitney *U* test; *n* = 10–12 tumors). (**B**) Representative BLI images from 27 days p.i. (**C**) At 31 d p.i. mice were injected with EdU for 2 h. Tumors were harvested and the percentage of EdU-positive cells was determined by flow cytometry. CD45-positive infiltrating immune cells were excluded. (Plotted are mean ± SEM. ^*^ *p* < 0.05, unpaired, two-tailed *t*-test; *n* = 4 tumors) (**D**) GFP fluorescence of control tumors or *TRIM52* knockdown tumors was determined by flow cytometry. (Plotted are mean ± SEM. ^**^p < 0.01, unpaired, two-tailed *t*-test; *n* = 9 tumors) (**E**) Schematic outline of *in vivo* competition experiment. U87MG cells expressing iRFP and harboring dox-inducible *TRIM52* shRNAs, or expressing BFP and harboring non-targeting shRNAs were mixed in a 70%–30% ratio. This cell mixture was pretreated with dox overnight or left untreated before subcutaneous implantation into flanks of athymic nude mice. Dox was provided in the drinking water for the duration of the experiment in the on-dox group. At 21 d p.i. single cell suspensions of harvested tumors were analyzed by flow cytometry for iRFP and BFP. (**F**) Representative FACS plots, and (**G**) quantification of CD45-negative cells are depicted (Plotted are mean ± SEM. ^**^ *p >* 0.01, ^***^ *p >* 0.001, paired two-tailed *t*-test; *n* = 6 tumors for no-dox animal, *n* = 12 for dox-treated animals).

Subsequently, these cells were subcutaneously implanted into the flanks of athymic nude mice (Figure 2A). Dox was provided in the drinking water to sustain GFP and shRNA expression, after which tumor growth was analyzed weekly by whole body BLI. At twelve days post-implantation (p.i.) Fluc levels could be reliably measured, yet there were no significant changes between cells expressing non-targeting and *TRIM52*-targeting shRNAs (Figure 2A). Subsequently, the non-targeted tumors robustly grew in size between day 18 and 31 p.i., which was paralleled by a strong increase in Fluc values. In contrast, the Fluc signal from *TRIM52*-targeted tumors was significantly lower at these time points (∼4-fold; Figure 2A, and 2B), indicating that *TRIM52*-knockdown diminishes tumor growth.

Next, proliferation of tumor cells was analyzed using the thymidine analogue 5-ethynyl-2’-deoxyuridine (EdU), which is incorporated into the DNA during S phase in proliferating cells. For this, tumor-bearing mice were injected with EdU for two hours, after which their tumor cells were analyzed by flow cytometry. In non-targeting shRNA expressing tumors, ∼13% of the tumor cells were labeled with EdU. However, in *TRIM52*-targeted tumors, only ∼7% of the cells were EdU positive, which is indicative of diminished cellular proliferation (Figure 2C).

Lastly, we determined by flow cytometry whether *TRIM52*-targeted tumors had reduced GFP -and thus shRNA- expression (Supplementary Figure 1A), indicative of out-selection of tumor cells with the strongest *TRIM52* knockdown as seen in cell culture (Figure 1C). Consistent with this, the MFI of *TRIM52*-targeted tumors was reduced by approximately 50% compared to their non-targeting counterparts (Figure 2D). Together, these results show that *TRIM52* ablation reduces tumor growth *in vivo*, which is consistent with our cell culture results (Figure 1B and 1C).

In a complementary experiment, cellular fitness of *TRIM52*-targeted and non-targeted U87MG cells was compared in a direct competition assay *in vivo*. For this, U87MG cells harboring dox-inducible *TRIM52*-targeting shRNAs were stably transduced with a lentiviral construct constitutively expressing iRFP, whereas non-targeted cells received an equivalent BFP vector (Figure 2E). These cells were mixed in a 70%:30% ratio, and subsequently implanted into athymic nude mice (Figure 2E). For this experiment, mice were either dox-fed to sustain shRNA expression, or not as controls.

Twenty-one days p.i., the percentage of iRFP-positive and BFP-positive cells in tumors was analyzed by flow cytometry and compared to percentages at the time of implantation (Figure 2F). CD45-positive infiltrating leukocytes from the mouse host were excluded from analyses, yet in all tumors a substantial fraction of all CD45-negative cells were neither iRFP, nor BFP positive. These cells are most likely murine fibroblasts which form part of the tumor, as well as U87MG cells which have lost iRFP or BFP expression.

In tumors derived from non-dox treated animals, the ratio of *TRIM52*-targeting cells and non-targeting shRNA cells resembled the situation before implantation with approximately 2.5 fold more iRFP-positive cells than BFP-positive cells (Figure 2F, 2G; left panel). In contrast, in tumors derived from dox-fed animals the majority of cells was BFP-positive (non-targeting shRNA cells), whereas only a small fraction of cells still expressed iRFP (*TRIM52* knockdown cells; Figure 2F, and 2G; right panel). This further demonstrates that upon *TRIM52* shRNA expression, U87MG cells have a selective disadvantage *in vivo*.

Taken together, these data show that TRIM52 is required for efficient tumor formation and cancer cell proliferation in a U87MG xenograft model. In line with this, cells with high *TRIM52*-targeting shRNA expression are preferentially lost during tumor formation, indicating that *TRIM52* ablation confers a selective disadvantage *in vivo*.

### *TRIM52* ablation increases the percentage of cells in G_0_ and G_1_ cell cycle stages, consistent with reduced growth speed

**Figure 3:**
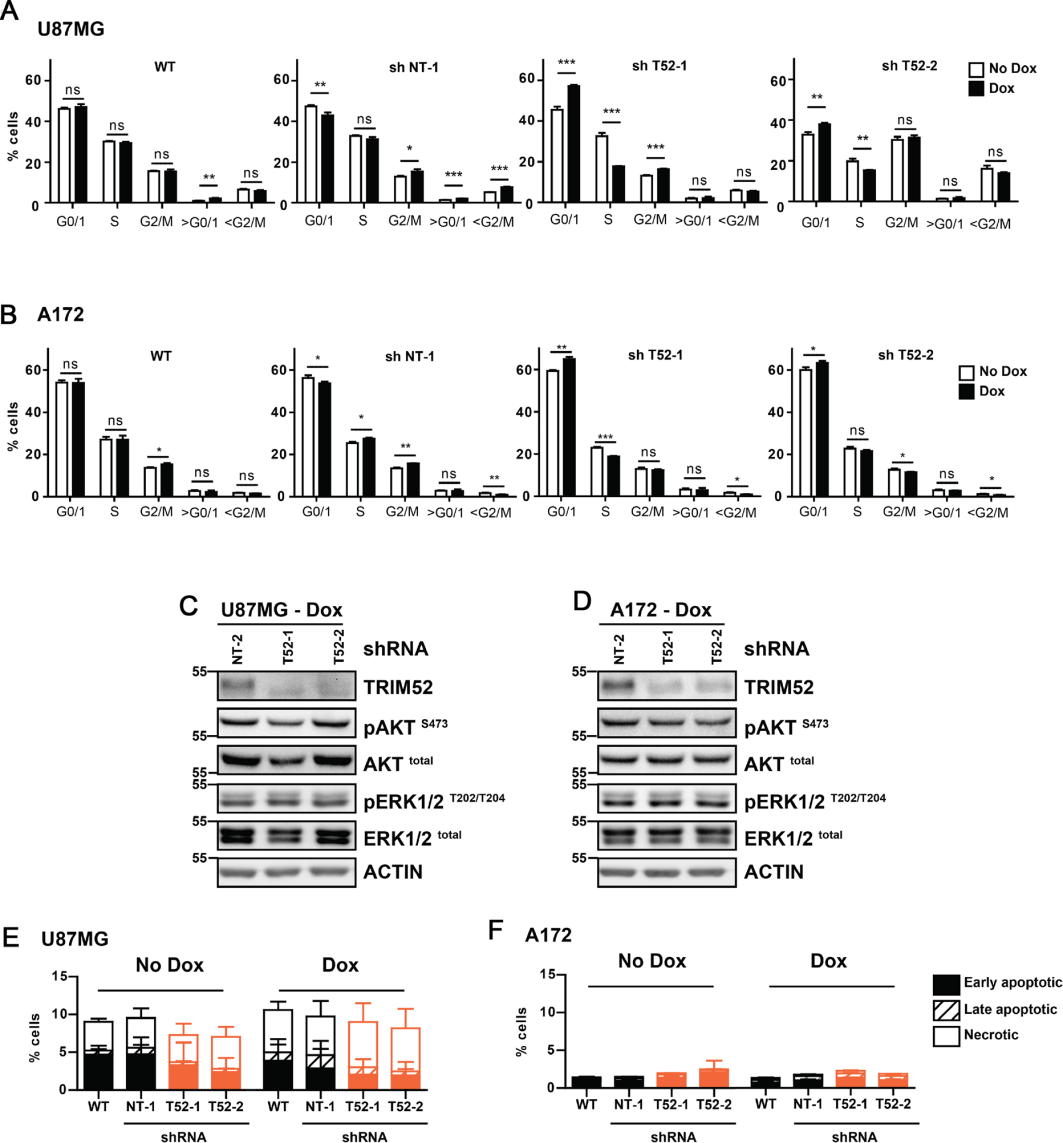
*TRIM52* knockdown increases the accumulation in G0/1 cell cycle phase (**A, B**) U87MG (**A**) or A172 (**B**) cells stably transduced with dox-inducible shRNA vectors were treated with dox, DNA was stained with propidium iodide, and DNA content was measured by flow cytometry. Data are representative of at least two independent experiments. Mean ± SD, *n* = 3; student’s *t*-test were calculated compared to non-dox treated cells per cell line: ^*^p > 0.05; ^**^p > 0.01; ^***^p > 0.001. (**C, D**) Western blot analysis of U87MG cells (C) and A172 cells (B) treated with dox. (**E, F**) U87MG (E) or A172 (F) cells as described in a. were stained with MitoStatus Red (polarized mitochondria), and propidium iodide (dead cells), and analyzed by flow cytometry. Percentages of early apoptotic (solid bars), late apoptotic (crossed bars) and necrotic cells (empty bars) were quantified. Data are representative of at least two independent experiments.

The reduced cell numbers of *TRIM52*-targeted U87MG and A172 cells in cell culture (Figure 1B) could have resulted from decreased cell proliferation, and/or increased cell death. To specifically test these two possibilities, first the effect of *TRIM52* knockdown on cell cycle distribution was investigated by widely-used cellular DNA content measurement [14]. *TRIM52* knockdown consistently increased the percentage of U87MG (Figure 3A, and Supplementary Figure 3A) and A172 (Figure 3B, and Supplementary Figure 3B) cells in G_0_/G_1_ and reduced the percentage in S-phase, whereas non-targeting controls did not introduce any substantial differences in cell cycle distribution. These data consistently indicate that *TRIM52* knockdown results in an increased fraction of cells in the G_0_/G_1_ cell cycle stages, indicative of a reduced proliferation speed. These data are consistent with the reduced EdU incorporation in *TRIM52*-targeted tumors *in vivo* (Figure 2C).

It has been reported that TRIM52 over-expression can activate NFκB [8, 15]. Since NFκB is one of the central cellular regulators of survival and proliferation [16], we investigated whether *TRIM52* knockdown affects NFκB activation. *TRIM52* knockdown in U87MG cells did not affect NFκB dual-luciferase reporter induction under steady-state conditions, nor did it significantly change TNF-induced reporter activation (Supplementary Figure 3C). In complementary experiments, NFκB activation was measured by phosphorylation, and subsequent degradation of its cytoplasmic inhibitor IκBα. In line with the results from the dual-luciferase reporter assay, the kinetics of TNF-induced IκBα phosphorylation (Supplementary Figure 3D; peak at 5 min.), and subsequent degradation (Supplementary Figure 3D; peak at 15 min.) were comparable in U87MG cells expressing a non-targeting control shRNA or *TRIM52*-targeting shRNAs. In sum, these data indicate that the effects of *TRIM52* ablation on cell proliferation are not the result of changes in NFκB activation.

Growth factor signaling dependent on ERK and AKT kinases is critical for cell cycle progression [17, 18]. Therefore, we investigated whether *TRIM52* knockdown affects activation by phosphorylation of these kinases, and thereby diminishes proliferation. *TRIM52* ablation in U87MG and A172 cells did neither reproducibly affect total levels of ERK and AKT, nor their phosphorylation (Figure 3C, 3D), suggesting that the observed growth defects are likely independent of these signaling pathways, or the result of defects down-stream of ERK and AKT.

Next, we set out to investigate whether in addition to a defect in proliferation, an increase in cell death contributed to the decreased cellular fitness of *TRIM52* knockdown cells. To this end, mitochondrial integrity was measured using Mitostatus Red dye. This dye accumulates in healthy mitochondria, yet is lost upon loss of inner mitochondrial membrane potential during apoptosis, necrosis, and oxidative stress. Under the same growth conditions during which *TRIM52* ablation reduced U87MG cellular fitness, both non-induced and dox-induced cells contained between 88–92% live (Mitostatus Red-positive) cells (Figure 3E, and Supplementary Figure 3E). Similarly, non-targeting and *TRIM52*-targeting shRNAs maintained the same 88–92% healthy cells in U87MG cells (Figure 3E). In A172 cells, similar results were obtained with ∼98% live cells under all observed conditions (Figure 3F, and Supplementary Figure 3F), indicating that differential cell survival did not substantially contribute to the observed differences in cellular fitness.

Glioblastoma cells often have go-or-grow characteristics; i.e. slowly proliferating cells are migrating more [19]. Since the two cell lines in which *TRIM52* ablation caused a growth disadvantage (U87MG and A172) are both of glioblastoma origin, we addressed whether *TRIM52* knockdown affects cell migration using a trans-well assay.

Serum addition in the bottom compartment of a Boyden chamber substantially increased cell migration from the upper compartment by ∼12 fold (Supplementary Figure 3G; from 5 cells to 60 cells per field of view), showing that the assay *per se* worked as expected. Nevertheless, cells expressing *TRIM52*-targeting shRNAs migrated to the same extent as their counterparts expressing non-targeting shRNAs in both the absence and presence of serum as a chemo-attractant (Supplementary Figure 3G), indicating that *TRIM52*-ablation did not affect cell migration.

In sum, we conclude from these combined data that *TRIM52*-ablation reduces cellular fitness in a cell context-dependent manner, which results predominantly from decreased cell cycle progression and hence proliferation.

### *TRIM52* knockdown induces global mRNA changes consistent with altered glucose metabolism responses, but cellular glycolysis and oxidative phosphorylation rates are not affected

To understand which cellular pathways are directly or indirectly affected by *TRIM52* knockdown and may underlie the observed proliferation differences, changes in mRNA abundance were compared by mRNAseq between U87MG cells expressing non-targeting, or *TRIM52*-targeting shRNAs. To this end, mRNA was harvested in biological duplicates at five days after shRNA induction by dox. This represents an early time point at which growth defects were detected (Figure 1B).

**Figure 4:**
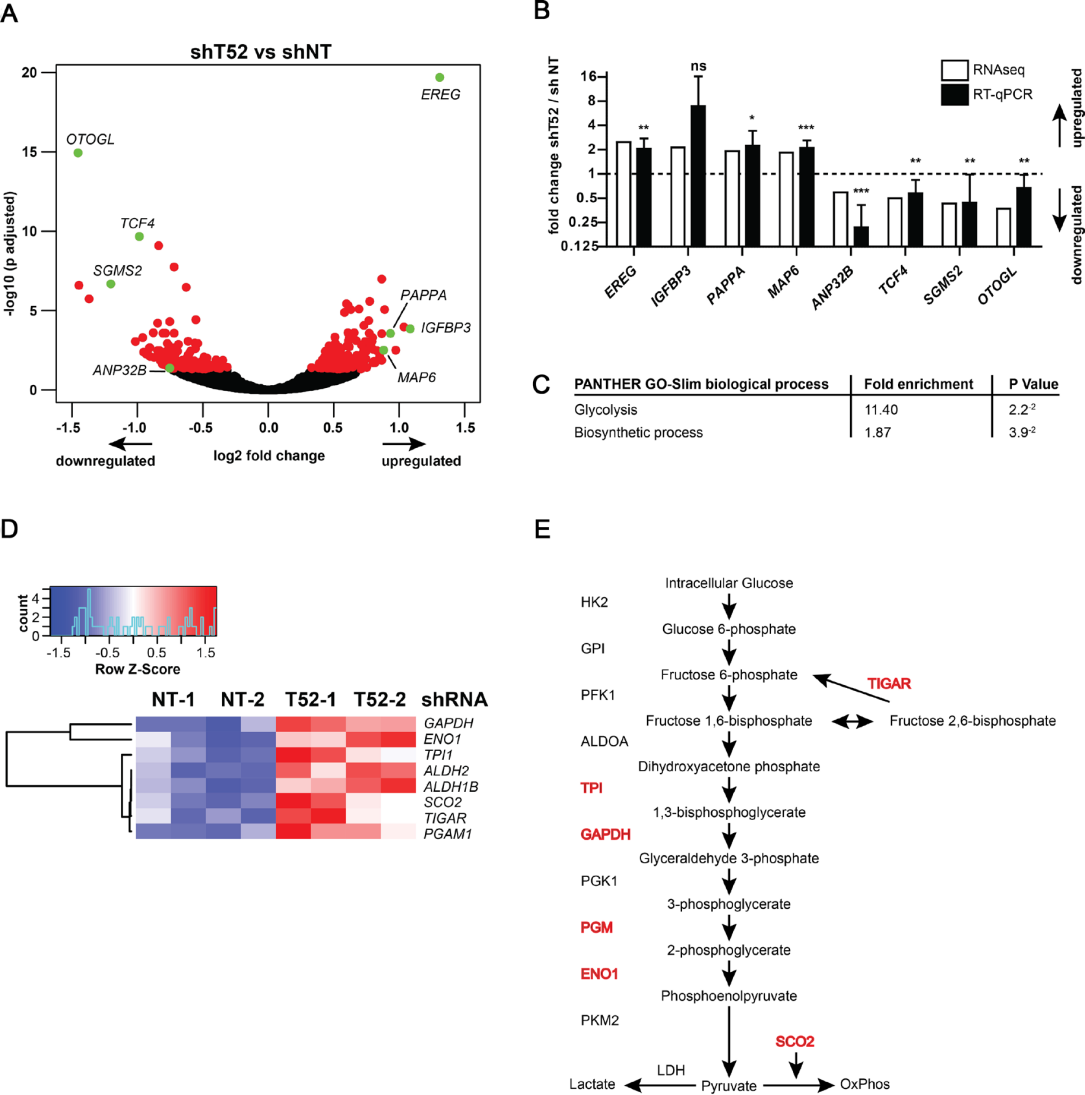
*TRIM52* knockdown cells have a glucose metabolism-related gene signature U87MG cells stably transduced with dox-inducible shRNA vectors were treated in duplicates with dox for 5 days; differential expression was analyzed by mRNAseq. (**A**) Volcano plot showing log2 fold change (*TRIM52* knockdown compared to non-targeting controls) plotted against –log10 adjusted *p*-value. Red dots indicate genes with *p >* 0.05; green dots indicate genes whose expression was subsequently analyzed by RT-qPCR. (**B**) RT-qPCR analysis in independent samples of a selected set of differentially regulated genes (indicated in green in panel a.). Gene expression was normalized to expression in non-targeting controls. Data represent samples from two independent experiments pooled; mean ± SD, *n* = 6; student’s *t*-test was performed relative to non-targeting controls: ^*^p > 0.05; ^**^p > 0.01; ^***^p > 0.001) (**C**) Gene Ontology analysis of all 278 differentially regulated genes analyzed by PANTHER GO-Slim; Bonferroni correction applied for *p*-value calculation to correct for multiple testing. (**D**) Heatmap of differentially regulated glucose metabolism genes. (**E**) Schematic of the canonical glycolysis pathway. Genes differentially expressed upon *TRIM52* knockdown are indicated in red.

Subsequent analysis was performed to identify mRNAs with significantly different expression in each replicate of both individual *TRIM52*-targeting shRNAs relative to their non-targeting counterparts. Using these conditions, a total of 278 mRNAs were identified as differentially expressed, of which 148 were up-regulated, and 130 were down-regulated in *TRIM52* knockdown cells (Figure 4A, and nd Supplementary Table 2).

Subsequently, a subset of differentially expressed mRNAs identified by mRNAseq was analyzed by RT-qPCR to determine reproducibility by an independent method. This validation set included genes with low, medium, and highest differential expression (Figure 4A, genes indicated in green). RT-qPCR analysis of the mRNAseq samples, and independently generated biological replicates showed reproducible differential expression of both up- and down-regulated genes (Figure 4B).

Next, Gene Set Enrichment Analysis (GSEA) was used to identify biological processes by Gene Ontology (GO) terms that were over- or under-represented in the set of identified genes. This identified “Glycolysis” (11.4-fold enrichment, *p =* 0.02) and “Biosynthetic Process” (1.9-fold enrichment, *p =* 0.04) as putatively affected biological processes (Figure 4C). Subsequent mapping of differentially expressed genes in the glucose metabolism pathway, indeed confirmed that genes encoding for enzymes involved in various stages of this pathway were up-regulated upon *TRIM52* ablation (Figure 4D, 4E, and Supplementary Figure 4).

It should be noted that none of these glucose metabolism genes were among the most-differentially expressed, and mostly clustered in the bottom 60% of increased genes. Similar GO analyses with only the most-changed genes, or separating up- and down-regulated genes did not identify any additional pathways (not shown).

Based on these observations, we next investigated whether *TRIM52* ablation affects glycolysis or oxidative phosphorylation, and thereby contributes to the reduced proliferation rate of these cells. To this end, extracellular acidification rates (ECAR) were determined as a measure of glycolysis, and oxygen consumption rates (OCR) as a measure of oxidative phosphorylation.

**Figure 5:**
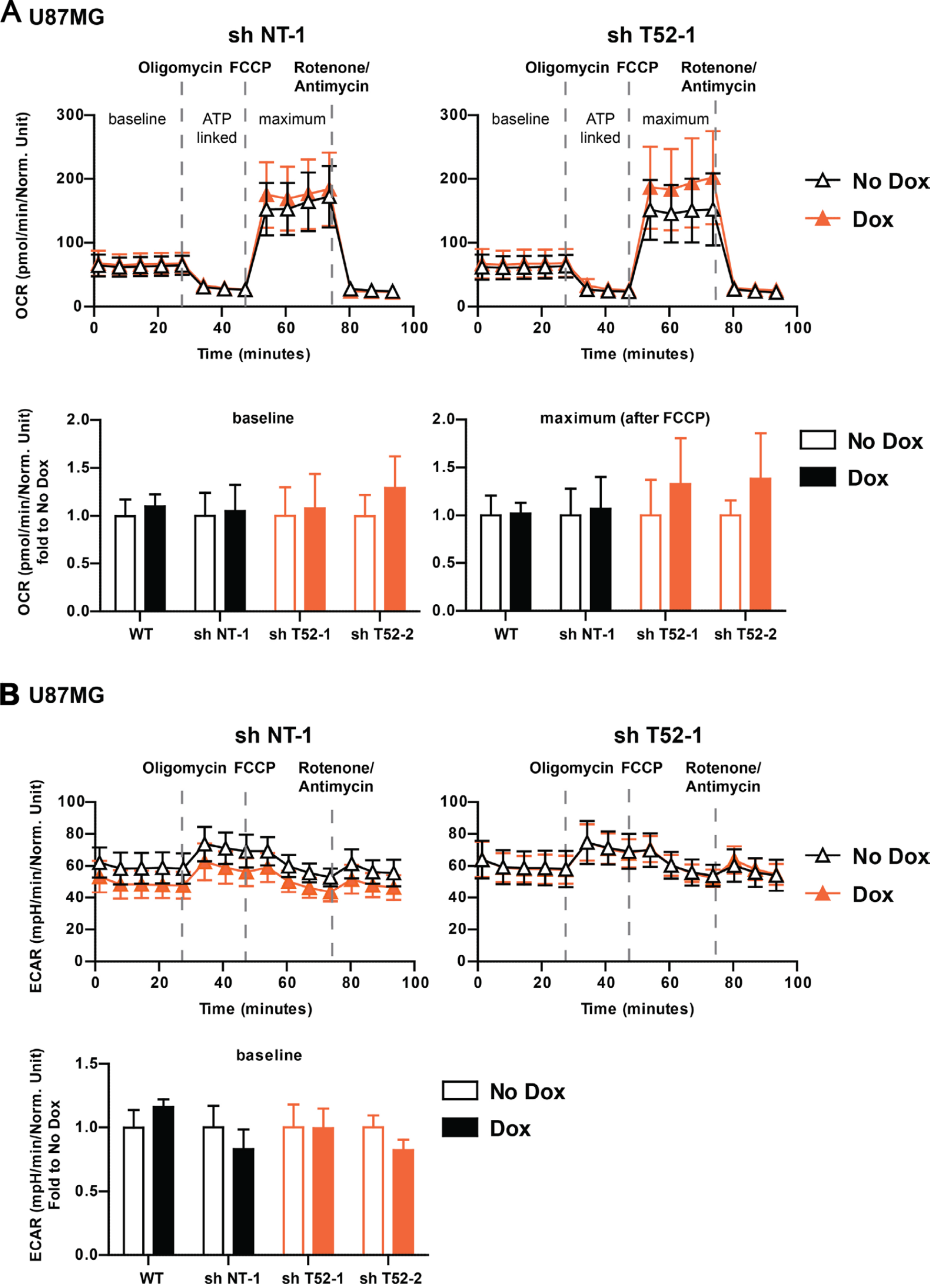
*TRIM52* knockdown does not affect Oxygen Consumption Rate (OCR) and Extracellular Acidification Rate (ECAR) (**A, B**) U87MG cells stably transduced with dox-inducible shRNA vectors were treated with dox for 4 days, and seeded into Seahorse bio-analyzer analysis plates. Measurements were carried out for up to 90 min. At indicated time points, oligomycin, FCCP or rotenone/antimycin A were injected into the wells. (A) Oxygen consumption rate (OCR) was measured and normalized to protein content. Fold basal (bottom left panel) and maximum (bottom right panel) OCR is displayed (*n* = 8–11). (B) Extracellular acidification rate (ECAR) was measured and normalized to protein content. Fold basal (bottom panel) ECAR is displayed (*n* = 8–11).

Under steady state growth conditions, which resulted in decreased U87MG growth by *TRIM52* knockdown, OCR were comparable in cells expressing non-targeting and *TRIM52*-targeting shRNAs (Figure 5A; time point 0). This indicates that *TRIM52* ablation did not result in substantial changes in basal respiration. Subsequently, different electron transport chain uncouplers were sequentially injected into the assay plate to measure whether ATP-linked respiration, maximal respiratory capacity, and respiratory reserve differed between cells (Figure 5A). Although the different uncouplers changed the OCR rates as expected, U87MG cells expressing different tested shRNAs all had comparable dynamically changed OCR rates (Figure 5A, and Supplementary Figure 5A), indicating that there are no major differences between them in oxidative phosphorylation.

Parallel ECAR determination of the different shRNA expressing cells, also indicated no major differences in basal lactate production (Figure 5B, and Supplementary Figure 5B), and hence glycolytic rates. Moreover, forced maximum glycolytic rates by blocking oxidative phosphorylation with oligomycin were also similar between all tested cell lines (Figure 5B, and Supplementary Figure 5B). From these combined data, we conclude that *TRIM52* knockdown does not substantially alter glycolysis or oxidative phosphorylation rates, and that it is unlikely that the observed changes in cellular proliferation upon *TRIM52* ablation stem from alterations in cellular energy production.

### *TRIM52* ablation reduces cell proliferation in a p53-dependent manner

We noticed that the GBM cell lines sensitive to *TRIM52* ablation (U87MG and A172; Figure 1B) are homozygous WT for *TP53* (the gene encoding the p53 tumor suppressor; Supplementary Table 1), whereas the insensitive lines are homozygous mutant (non-functional; Supplementary Table 1). Since activated p53 can inhibit cell cycle progression [28], we reasoned that *TRIM52* ablation may reduce proliferation in a p53-dependent manner. This was further supported by the observation that two well-characterized p53-dependent genes (*TIGAR* and *SCO2*; Figure 4D) were up-regulated by *TRIM52* knockdown.

**Figure 6:**
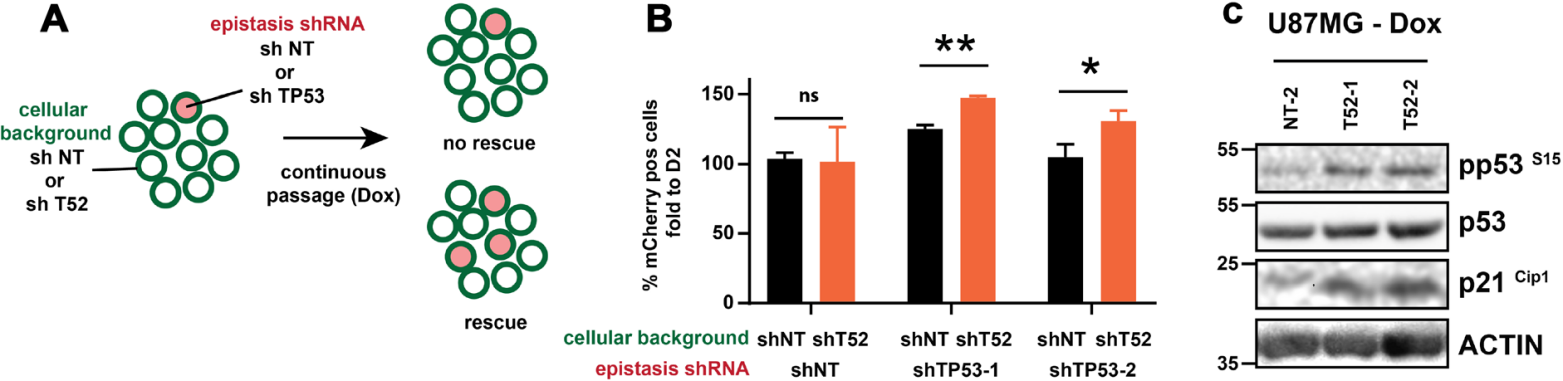
*TP53* ablation rescues proliferation of *TRIM52* knockdown cells (**A**) Overview of experimental setup. (**B**) U87MG cells stably harboring dox-inducible *TRIM52*-targeting or non-targeting shRNAs were stably transduced at low transduction efficiency with vectors expressing mCherry as well as a non-targeting, or one of two *TP53*-targeting shRNAs. Cells were cultured in the presence of dox for 11 days, after which the percentage of mCherry-positive cells was analyzed by flow cytometry. Values are plotted relative to the percentage mCherry positive cells at day 2 post-induction with dox. (**C**) U87MG cells stably harboring dox-inducible *TRIM52*-targeting or non-targeting shRNAs were treated with dox. At day 7 post-induction whole cell lysates were analyzed by WB using the indicated antibodies.

To investigate whether p53 is required for reduced proliferation in *TRIM52* knockdown cells, we performed a competition epistasis experiment in which *TP53* was simultaneously targeted in the same cells as *TRIM52* (Figure 6A). This was achieved by transducing the *TRIM52* knockdown cells with an mCherry-expressing retroviral vector encoding *TP53*-targeting or non-targeting shRNAs. Subsequently, *TRIM52* knockdown was induced by dox (which also drives GFP expression; Supplementary Figure 1A), after which the relative abundance of cells with the various shRNAs was analyzed by flow cytometry.

As expected, expression of a non-targeting shRNA in *TRIM52*-knockdown cells or control cells did not significantly change their abundance in the mixed culture (Figure 6B). In contrast, ablation of *TP53* with two independent shRNAs significantly increased their proportion in the cell pool relative to *TP53* knockdown in cells in which *TRIM52* was not targeted (Figure 6B). Together, these results indicate that *TRIM52* ablation decreases U87MG proliferation in a p53-dependent manner. In agreement with this observation, WB analysis showed that the level of activated p53 (pSer15) and one of its major targets, the cyclin-dependent kinase inhibitor p21^Cip1^, were increased by *TRIM52* ablation (Figure 6C), whereas the total levels of p53 remained unchanged.

In conclusion, the data in this study show that TRIM52 expression is required for optimal cell cycle progression in U87MG and A172 glioblastoma cell lines, whereas it is dispensable for optimal growth of various other tested cancer cells lines. This suggests that this recently evolved protein is required for optimal proliferation depending on the genetic context of individual cancer cell lines. We identified p53 as such a factor, although it remains to be determined whether other factors affect TRIM52-dependent phenotypes, and how TRIM52 controls proliferation at the molecular level.

## DISCUSSION

For optimal proliferation, evasion of cell death and adaptation to the tumor-environment, many cancer cells rely on factors otherwise dispensable for cell growth under physiological conditions [12]. Here we specifically showed that TRIM52 provides a fitness advantage in two glioblastoma cell lines, but not in several other cancer cell lines.

The fact that the *TRIM52* gene has been acquired recently in evolution, and again was lost or pseudogenized in many mammals suggests that it likely does not play a role in any essential processes in normal cells. However, this has not been formally tested, and will be experimentally addressed in future studies. Given that both cell lines that did have a proliferation phenotype (U87MG and A172) are brain-derived, it will be important to include various primary human brain cell types in these experiments. Moreover, one of the noteworthy points of *TRIM52* evolution is that it was acquired in mouse and rat independently of the primate *TRIM52* gene. Hence, it will be of interest to test whether ablation of e.g. mouse *Trim52* affects glioblastoma cell proliferation.

Since the *TRIM52* gene arose as a partial gene duplication of *TRIM41*, it could be that their encoded proteins regulate each other’s expression or function. In fact, yeast-two-hybrid data has suggested that both proteins can physically interact [20]. Our analyses in *TRIM52* knockdown cells demonstrated that *TRIM41* mRNA expression remained unaltered. This indicates that the observed proliferation effects did not result from influencing *TRIM41* mRNA expression. Moreover, TRIM41 has been reported to mediate degradation of various PKC isoforms, including the widely expressed PKCα [5], which themselves regulate cancer cell proliferation under various conditions. Our preliminary data suggest that *TRIM52* knockdown does not affect PKCα levels, indicating that the observed proliferation differences are likely independent of functional cross-talk with TRIM41, although at this point we cannot rule out effects on other, undiscovered TRIM41-dependent processes.

In knockdown studies it is important to ensure that observed phenotypes are dependent on shRNA expression, and specific for the intended mRNA target. In this study dox addition *per se* minimally influenced the experimental read-out in all assays, while specifically reducing proliferation in cells harboring *TRIM52*-targeting constructs, but not non-targeting shRNAs. Moreover, two independent shRNAs, targeting different parts of the *TRIM52* mRNA both yielded the same phenotype, attesting to their specificity and making it unlikely that the observed effects are the result of off-targets.

Currently it remains unclear i) how TRIM52 influences cell cycle progression at a molecular level, and ii) whether TRIM52 plays a role in non-cancer cells. The results from this study have provided the means to address these questions in the future.

Proliferation in many glioblastoma lines is highly dependent on EGFR signaling through AKT and ERK pathways. Although these signaling molecules regulate growth factor dependent proliferation, they also control a plethora of other metabolism and cell death dependent functions. One of the noteworthy differences between the U87MG and A172 cells, compared to the other tested glioblastoma lines, is that they both are homozygous WT for *TP53* (the gene encoding p53), while carrying homozygous inactivated *PTEN* alleles. Both of these genes have been implicated as important factors regulating energy metabolism through mTORC complexes, growth factor signaling, and oncogene expression in many tumors [21–23]. As such, they are likely candidates to contribute to the cell-specific effect of *TRIM52* knockdown on proliferation. In line with this notion, simultaneous *TP53* knockdown rescued proliferation in *TRIM52* knockdown cells, indicating that *TRIM52* knockdown decreased proliferation in U87MG cells in a p53-dependent manner.

However, *TRIM52* ablation in LNCaP prostate cancer cells (also wt *TP53*, and mutant *PTEN*) did not affect proliferation in these cells. This indicates that a combination of homozygous WT p53 and mutant PTEN alleles may not be sufficient to explain the lack of a proliferation phenotype in this cell line, and suggests that other factors are involved.

It is noteworthy that of the tested cell lines, the two with a proliferation defect in *TRIM52*-ablated cells are of glioblastoma origin, indicating TRIM52 may regulate proliferation in a genetic context that is glioma-, or brain-specific. In this regard, p53 and PTEN have been shown to determine glioma stem/progenitor cell renewal and differentiation [24], which could ultimately affect proliferation speed. Many glioblastoma cell lines (e.g. U87MG) can form neurospheres under specific culture conditions [25], yet the percentage in regular culture conditions as used in this study will predominantly favor non-neurosphere (two-dimensional) growth. We have not observed any obvious differences in the low percentage of spontaneous neurosphere formation in *TRIM52*-knockdown cells at this point, suggesting that differences in neurosphere formation may not underlie the proliferation defect. Nevertheless, we plan to investigate this in follow-up studies under controlled cell culture conditions favoring neurosphere formation.

In addition to differentiation, the effect of *TRIM52* ablation on proliferation could also result from differences in cells that have exited cell cycle (e.g. quiescent cells in G_0_). Our analysis showed that *TRIM52* knockdown increases the percentage of cells in G_0_/G_1_, but it was not possible with the used staining to distinguish between these two cell cycle states. Attempts to distinguish between these two, by staining for the widely-used proliferation marker Ki67, have been inconclusive. Thus, our analyses indicate that thus far there is no clear correlation between *TRIM52* knockdown and changes in quiescence.

It is important to mention that TRIM52 function had previously only been addressed in over-expression experiments, which had suggested a role of TRIM52 in NFκB activation. In our experiments, *TRIM52* knockdown affected NFκB activation neither under resting, nor cytokine-stimulated conditions, indicating that differential NFκB activity does not underlie the proliferation defect in *TRIM52*-knockdown cells. Although this could mean that TRIM52 only regulates NFκB activity at high expression levels, it could also suggest that this may be cell-type specific, or that functional redundancy exists.

Lastly, we set out to identify pathways affected by *TRIM52* knockdown using mRNAseq. Overall, *TRIM52* ablation resulted in relatively moderate changes in gene expression. Nevertheless, both manual curation and automated GO analysis consistently identified various glucose metabolism genes to be up-regulated in both *TRIM52*-targeted cell lines, compared to non-targeting controls. Nevertheless, we could not detect differences in actual oxidative phosphorylation or glycolysis, suggesting that *TRIM52* ablation does not substantially alter these processes, and hence are unlikely to be responsible for the observed differences in proliferation.

One should keep in mind that the changes in glucose metabolism genes may represent an indirect effect that is independent of the altered proliferation, or is not reflected at the protein level. Moreover, it is important to note that neither the rate limiting enzyme in glycolysis (Phosphofructokinase-1 [26]), nor any of the glucose transporters [27] (GLUT) were differentially regulated by *TRIM52* knockdown. This may indicate that the glucose metabolism gene signature could be reflective of changes in an upstream pathway or factor controlling their expression, rather than a cause for altered proliferation.

In line with our data indicating that *TRIM52* knockdown diminishes proliferation in a p53-dependent manner (Figure 6), many metabolic genes are controlled by p53. In particular the up-regulated *TIGAR* and *SCO2* mRNAs are specific p53 targets [28], suggesting that part of the differential mRNAseq profile may stem from p53 dysregulation. Future work will be focused on elucidating the effect of TRIM52 on other key transcription factors controlling these glycolysis genes [29], and the effects of *TRIM52* ablation in conditions of external p53 activation such as by the DNA damage response [28]. Together, these approaches will ultimately contribute to unraveling how TRIM52 controls proliferation at the molecular level.

In summary, here we report that TRIM52 is required for optimal proliferation in a cell context-dependent manner. Our data show that in some cell lines p53 may be an important factor determining TRIM52-dependent biological output. Although the exact molecular mechanism by which TRIM52 does so has remained elusive, this study shows that this evolutionary non-conserved protein could be a target for combinatorial gene disruption approaches in certain cancer types. Moreover, these data have provided the first steps for more detailed work to understand how TRIM52 controls proliferation at a molecular level.

## MATERIALS AND METHODS

### Cells

U87MG, A172 and U251 cells were a kind gift from Prof. Wolfgang Sattler (Medical University Graz); HCT116 cells were a kind gift from Prof. Manuela Baccarini (MFPL, University of Vienna); T98G cells were a kind gift from Dr. Ulrich Elling (IMBA, Vienna); DU145 cells were a kind gift from Prof. Gerda Egger (Medical University of Vienna); K562 cells were a kind gift from Dr. Andreas Brachner (University of Vienna). K562 and DU145 cell lines were cultured in RPMI (Sigma, R7388) supplemented with 10% fetal calf serum (FCS; Sigma, F7524) and 1% Penicillin-Streptomycin (Sigma, P4333). All other international cell lines were cultured in high glucose DMEM (Sigma, D6429) supplemented with 10% FCS and 1% Penicillin-Streptomycin. Patient-derived cell models were established from surgical specimens as previously described [30]. All primary cell models (BTL53, BTL1376, BTL1529, BTL2175, BTL2176, BTL2177, VBT12, VBT25, VBT72) were derived from glioblastoma multiforme (GBM) specimens [13]. After establishment, primary cell lines were cultured in RPMI 1640 medium supplemented with 10% FCS without antibiotics. The study was approved by the local ethic committees and all patients have given written informed consent for further use of the tumor material. Cerebral organoids were a kind gift from Veronica Krenn and Jürgen Knoblich (IMBA, Vienna) and were generated as previously described [31]. All cells were cultured at 37° C and 5% CO_2_ in a humidified incubator.

### Generation of stable cell lines by lentiviral transduction

For lentivirus-like particle (VLP) production, HEK-293T cells were seeded at ∼20% confluency into 6-well clusters. The next day, cells were transfected as previously described [32] with 454.5 ng pSPAX2-GagPol plasmid, 454.5 ng mini genome and 91 ng VSV-G plasmid using polyethyleneimine (PEI) as transfection reagent (Polysciences, 23966-2). Two days post transfection, recipient cells were seeded to ∼20% confluency into 6-well clusters. Three days after transfection, VLP-containing supernatants were harvested from producer cells and filtered through a 0.45 µM filter (Sarstedt, 83.1826). Supernatants were diluted 1:5 with full growth media containing 8 µg/ml Polybrene (Millipore, TR-1003-G). Media from recipient cells were replaced with VLP-containing media. Three days after transduction, cells were selected using 3 µ/ml Puromycin (Fisher, 10296974) for at least three days and kept under Puromycin selection.

### shRNA mediated knockdown

The retroviral LENC plasmid for stable shRNA expression was a kind gift from Johannes Zuber (IMP, Vienna Austria) and has been previously described [13]. pLT3-GEPIR plasmid for dox-inducible shRNA expression (a kind gift from Johannes Zuber (IMP, Vienna Austria)), as well as cloning strategy was described previously [13]. In brief, shRNAs were ordered as 97 bp oligos (IDT) which contain the optimized miR-E backbone as well as gene-specific shRNA regions. DNA was amplified by standard PCR using the following primers (F: 5′- tacaatactcgagaaggtatattgctgttgacagtgagcg-3′; R: 5′- ttagatgaattctagccccttgaagtccgaggcagtaggca – 3′). PCR products were cloned into pLT3-GEPIR plasmid using EcoRI (NEB) and XhoI (NEB) restriction enzymes and successful cloning was confirmed by Sanger sequencing (Eurofins genomics), using 5′-tgtttgaatgaggcttcagtac-3′ primer. shRNA target sequences were: *TRIM52-1*: 5′-atacgatgaggacgaagatgaa-3′; *TRIM52-2*: 5′-ccaagaccaagatgacgatgaa-3′; NT-1 (targeting Renilla luciferase): 5′-taggaattataatgcttatcta; NT-2 (previously described in [33]): 5′-cctaaggttaagtcgccctcgc-3′; *TP53*-1: 5′-actggaagactccagtggtaat-3′; *TP53*-2: 5′- tggaggatttcatctcttgtat-3′. To induce shRNA expression, cells were treated with 2 µg/ml dox (Sigma-Aldrich; D9891) for 4 days unless stated otherwise.

### Animal studies

All animal experiments were carried out in agreement with the ethical animal license protocol in accordance with the current laws of Austria, approved by the Bundesministerium für Wissenschaft und Forschung, Austria (Permit number BMWFW 66.015/0010-WF/v/3b/2017). 6–12 week-old, sex-matched in-house bred MF-1 Nu/Nu mice were used for animal experiments. To track differences in tumor growth, 2.5 × 10^6^ tumor cells pre-treated with dox for 24 h were injected subcutaneously in a 1:1 mixture of PBS/Matrigel (Corning, #356237). For the *in vivo* competition assay, 3.5 × 10^6^ dox-pretreated cells were injected. To sustain shRNA-mediated knockdown, mice received dox (2 mg/ml; Sigma, D9891) in the drinking water. Tumor formation and outgrowth was tracked by bioluminescence imaging (BLI) of luciferase-labelled tumor cells. In brief, isoflurane-anesthetized mice were injected with D-luciferin (150 mg/kg) and imaged with an IVIS spectrum Xenogen machine (Perkin Elmer). Bioluminescence analysis was performed using Living Image software, version 4.4.

### Flow cytometry analysis of tumors

To generate single cell suspensions, tumors were minced and digested with 1mg/ml collagenase A (Roche, 10103586001) in PBS, for 1.5 h at 37° C. Cells were washed with PBS containing 5mM EDTA and 1% bovine serum albumin (BSA), dislodged and passed through a 100 µM cell strainer (Corning, 431752). For subsequent flow-cytometry analysis, cells were treated with anti-mouse Fc-block CD16/32 antibody (2.4G2 BD) in PBS containing 5mM EDTA and 1% BSA for 10 min on 4° C. Cells were stained with murine CD45-PeCy7 antibody (BD 30-F11 1:500) for 30 min. Expression of GFP, iRFP and BFP was analyzed on CD45 negative cells. For experiments involving EdU, mice were injected intraperitoneally with 50 mg/kg EdU, after 2 hours tumors were collected, single cell suspensions were generated as described above and further processed according to the manufacturer’s protocol (Click-iT Plus EdU Flow cytometry Pacific Blue Assay kit, Invitrogen #C10636). Samples were analyzed on a FACS Fortessa (BD Biosciences).

### Antibodies

For Western blots the following antibodies were used at the indicated dilutions. Actin (Sigma, A2103, 1:1000); AKT (CST, 9272, 1:1000); pAKT-S473 (CST, 9271, 1:1000); ERK1/2 (CST, 9102, 1:1000); pERK1/2-T202/T204 (CST 9101, 1:1000); HA-epitope (Abcam, ab137838, 1:1000); IκBα (CST, 4812, 1:1000); pIκBα-S32/36 (CST, 9246, 1:1000) ); p21^Cip1^ (Abcam, ab7960, 1:1000); p53 (CST, #2524, 1:1000); pp53-S15 (CST, #9284 P, 1:1000); TRIM52 (clone A4; Santa Cruz Biotechnologies sc-398954, 1:250); anti-rabbit-IgG-HRP (CST, 7074S); anti-mouse IgG-HRP (CST 7076S, 1:4000).

### Cell counting

Cells were seeded at 1 × 10^5^ cells/well in 6-well clusters unless indicated otherwise, in media containing dox (2 µg/ml) or not. At indicated time points, supernatants from cells were harvested to collect non-adherent cells, whereas adherent cells were washed once with PBS and trypsinized (Sigma T4049). Cells were resuspended in their cognate supernatants. Then, cells were counted using a CASY cell counter (Innovatis, TTC-2KA-2037).

### Apoptosis and viability determination

Cells were co-stained with MitoStatus Red (BD, 564697) and Propidium Iodide (BD 556463). MitoStatus Red was added at a final concentration of 66.6 nM to cells and incubated at 37° C/5% CO_2_ in the dark for 30 min. Supernatants were collected; adherent cells were trypsinized and resuspended in their cognate supernatants to retain non-adherent cells. Cells were washed twice with FACS buffer (PBS, 1% BSA, 5 mM EDTA) and resuspended in 200 µl FACS buffer containing 1.25 µg/ml Propidium Iodide and incubated for 10 min. at 4° C in the dark. Cells were analyzed by flow cytometry using the APC (MitoStatus red) and PE (PI) channels.

### Cell cycle analysis

Stable inducible knockdown cells were treated with dox for 4 days. Subsequently, cells were seeded at 5 × 10^5^ cells/10 cm dish and were allowed to proliferate for another 48h. Subsequently, cells were trypsinized and resuspended in serum-free DMEM. Cells were washed once and resuspended in 100 µl cold PBS. Cells were fixed and permeabilized by dropwise addition of 300 µl ice-cold ethanol (absolute) and incubated at –20° C for at least 1 h. Cells were spun and washed once with PBS and resuspended in staining buffer (PBS containing 1% BSA, 5 mM EDTA, Propidium Iodide (SBT, sc-3541), 100 µg/ml RNAseA (Sigma, R5503)) and incubated at room temperature for 10 min to allow RNA degradation. PI fluorescence was measured in the PE channel (linear) of a FACS Fortessa (BD). Data was analyzed using FlowJo v10.0.7 using the Watson pragmatic model [34].

### Competition assay

Competition assays were performed as previously described [35]. In brief, cells stably transduced with pLT3-GEPIR plasmids containing *TRIM52*-targeting or non-targeting shRNAs (80%) were mixed with WT cells (20%), media was supplemented with dox (2 µg/ml). The following day, GFP fluorescence intensity and percentage GFP-positive cells were measured by flow cytometry. Cells were split every two days in the presence of dox in a 1:2 ratio (U87MG) or 1:5 ratio (HeLa), respectively, and GFP signal was measured by flow cytometry. For the *TP53* epistasis assay, U87MG cells stably transduced with pLT3-GEPIR plasmids containing *TRIM52*-targeting or non-targeting shRNAs were additionally transduced at low percentage with retroviruses stably expressing mCherry as well as a non-targeting or one of two *TP53*-targeting shRNAs. Cells were cultured in the presence of dox (2 µg/ml) for eleven days, after which the percentage of mCherry-positive cells was analyzed by flow cytometry.

### Metabolic flux measurements

Oxygen consumption rate (OCR) and extracellular acidification rate (ECAR) were determined on a Seahorse XFe96 Analyzer (Agilent) using the Seahorse XF Cell Mito Stress test kit (Agilent, 103015-100). In brief, U87MG cells were treated with dox (2 µg/ml) for 4 days to induce knockdown. Subsequently, cells were seeded in DMEM (Sigma, D6429) supplemented with 10% FCS and 1% Penicillin-Streptomycin at 1 × 10^5^ cells/well into Seahorse XF cell Culture Microplates (Agilent 102601-100) and cultured overnight. One hour prior to the assay, media was changed to XF Base Medium (Agilent 102353-100) containing glucose (10 mM), sodium pyruvate (1 mM) and L-glutamine (2 mM). The assay was run according to the manufacturer’s instructions and OCR and ECAR were measured for 95 min. After 27, 47 and 73 min, Oligomycin (1 µM), Carbonyl cyanide-p-trifluoromethoxyphenylhydrazone (FCCP, 1 µM) and Rotenone/Antimycin A (500 nM) were subsequently injected into wells, respectively. Raw data was analyzed using Wave Desktop Software (Agilent, version 2.0) and exported and graphed in GraphPad Prism (GraphPad Software, version 7.0a).

### mRNAseq analysis

U87MG cells harboring dox-inducible non-targeting shRNAs or TRIM52-targeting shRNAs were treated with dox (2 µg/ml) in biological duplicates for 5 days. GFP-positive cells were sorted by flow cytometry using a FACS Aria (BD). 1 × 10^6^ cells were lysed using Trizol reagent (Fisher 15596-018) and total RNA was isolated as recommended. RNA quality was determined by NanoDrop (Thermo Fisher) and Bioanalyzer chip (Agilent, 5067-1511). Subsequently, 1µg of RNA was used for polyA enrichment and Next Generation Sequencing (NGS) library preparation using a SENSE mRNA-Seq library prep kit V2 (Lexogen, 001.24) according to the manufacturer’s recommendations. The library was subjected to high-throughput sequencing single-end 50 bp on an Illumina HiSeq 2500 platform at the NGS unit of the Vienna BioCenter Core Facilities (VBCF) (http://vbcf.ac.at). The reads obtained from the instrument were base called using the instrument manufacturer’s base calling software. Demultiplexing was performed using Illumina2bam (http://gq1.github.io/illumina2bam/). The reads were aligned against the *Homo sapiens* reference genome (UCSC hg19 release) with STAR version 2.5.1b using the 2-pass alignment mode and reads mapping to ribosomal RNAs were removed. We obtained more than 80% uniquely mapped reads in each sample. After alignment, the reads were associated with known genes based on annotations derived from UCSC and the number of reads aligned within each gene was counted using the HTSeq tool (version 0.5.4p3). Counts were normalized using the TMM normalization method of the edgeR R/Bioconductor package (R version 3.3.1, Bioconductor version 3.3). Differential expression analysis was performed using Deseq2 (v. 1.10.1) on data normalized to effective library size using an adjusted *p*-value cutoff of 0.05 as previously described [36]. mRNAseq data are available under GEO accession number GSE107932.

### Statistical analyses

GraphPad Prism was used to calculate statistics.Two-tailed student’s *t*-test, two-tailed Mann Whitney *U* test, and one-way ANOVA were performed as indicated in the figure legends. In this study, a *p*-value of < 0.05 was considered statistically significant. All values are represented as mean ± SD unless otherwise indicated.

## SUPPLEMENTARY MATERIALS FIGURES AND TABLES







## Abbreviations

ATP: Adenosine triphosphate
AKT: AKT Serine/Threonine Kinase 1
BFP: Blue fluorescent protein
ECAR: Extracellular acidification rate
GFP: Enhanced green fluorescent protein
ERK: Extracellular signal-regulated kinase
Dox: Doxycycline
GBM: Glioblastoma multiforme
GO: Gene ontology
GSEA: Gene Set Enrichment Analysis
IκBα: Inhibitor of κB
iRFP: Infra-red fluorescent protein
lncRNA: Long non-coding RNA
mAb: Monoclonal antibody
NFκB: Nuclear factor κB
NS2A: Non-structural protein 2A
NT: Non-targeting shRNA
OCR: Oxygen consumption rate
PI: Propidium iodide
PKC: Protein kinase C
PTEN: Phosphatase and tensin homolog
RING: Really Interesting New Gene
RT-qPCR: Reverse transcription, quantitative polymerase chain reaction
shRNA: Short-hairpin RNA
T52-1/T52-2: *TRIM52*-targeting shRNA
TNF: Tumor necrosis factor
TP53: Tumor protein p53
TRIM: Tripartite motif protein
UTR: Untranslated region
