# Supplementary material for: Human tripartite motif protein 52 is required for cell context-dependent proliferation
Source: Oncotarget. 2018 Feb 5;9(17):13565–81. doi: 10.18632/oncotarget.24422 (PMC5862599; doi:10.18632/oncotarget.24422)
Supplement: Supplementary file 1 [file oncotarget-09-13565-s001.pdf]

Human tripartite motif protein 52 is required for cell context-dependent proliferation

SUPPLEMENTARY MATERIALS

Western blot analysis

1×10^6 Cells were lysed directly in 100 µl denaturing sample buffer (contains 1.6 M Urea, 1 M SDS, 3.5 M β-Mercaptoethanol). Samples were boiled for 10 min at 95° C, DNA was sheared. 20 µl sample were loaded onto 10% Polyacrylamide gels for SDS page. Following transfer to nitrocellulose membranes, blots were blocked in TBS containing 5% BSA for one hour. Blots were incubated with primary antibodies overnight at 4° C. Subsequently, blots were washed and incubated with HRP conjugated secondary antibodies for 1 h. Bands were visualized with Western Blotting Luminol Reagent (Santa Cruz, sc-2048). In case low signals were detected, Luminol reagent was supplied with 10% Super Signal West FEMTO max. sensitivity reagent (Fisher, 10187393). Detections were carried out on a ChemiDoc Touch Imaging System (BioRad) and relative protein levels were quantified using Image Lab (BioRad).

Transfections

Cells were seeded at low density (~20% confluency). DNA was mixed with Polyethylenimine (Polysciences, 23966-2) at a ratio of 1:3 (µg DNA/µg PEI), together with OptiMEM media (Fisher, 10592693). Mixes were incubated for 30 min at room temperature; subsequently, mixes were distributed on cells. Cells were harvested 48 h after transfection, unless stated otherwise.

NFkB luciferase assay

U87MG glioblastoma cells stably transduced with doxycycline inducible *TRIM52*-targeting or non-targeting shRNA constructs were treated for 4 days with 2 µg/ml doxycycline. Subsequently, cells were seeded 1:3 in M24 well clusters and the following day transfected with 200 ng pRL-TK and 200 ng pNFkB-Luc plasmids. Eighteen hours post-transfection, cells were stimulated with 10 ng/ml human TNFα (Peprotech; 300-01A) for 24 h and subsequently lysed in Passive Lysis Buffer (Promega; E194). Firefly- and Renilla luciferase activity was measured using Promega Dual luciferase assay reporter system (E1910) according to the manufacturer's recommendations, on a Synergy H1 plate reader (BioTek).

Transwell assay

U87MG glioblastoma cells stably transduced with doxycycline inducible *TRIM52*-targeting or non-targeting shRNA constructs were treated for 4 days with 2 µg/ml doxycycline. Subsequently, a total of 1×10^5 cells was seeded in DMEM medium without serum into 12 well TC inserts, pore size 8 µm (Sarstedt; 83.3931.800). The bottom compartment contained DMEM medium, supplemented with 10% FCS. After an incubation of five hours, inserts were removed, cells that did not migrate were removed using a cotton swab and migrated cells were fixed with Methanol and subsequently stained with Propidium Iodide (santa cruz sc-3541). For each well, six random fields of view were imaged using a Zeiss Axiovert 200 M (Zeiss), images were processed using Zen Blue (Zeiss) and cell numbers were obtained by using the Analyze Particles plugin from Fiji/ImageJ.

RNA isolation, cDNA generation and RT-qPCR

Total RNA was isolated using Trizol reagent (Fisher 15596-018) according to the manufacturer's recommendations. Contaminating DNA was digested using Turbo DNAse (Fisher 10722687). After re-precipitation of the RNA to remove divalent cations, DNAse was heat-inactivated. Sample purity was confirmed by NanoDrop (Thermo Fisher). RNA was reverse transcribed using Thermo Fisher RevertAid H Minus Reverse Transcriptase (EP0451) and Applied Biosystems random primers (4319979) according to manufacturer's recommendations. 2 × qPCR mastermix contained: 20 mM Tris pH 8.5, 100 mM KCl, 0.3% Triton X-100, 4 mM MgCl, 400 µM dNTPs (Promega U1515), 400 mM Trehalose (Sigma T9531), 5% Formamide (Sigma 47670) , 0.01% SYBR Green (Fisher 10207252), 50 u/ml Taq-polymerase (Promega M7848). qPCR was performed in technical triplicates using 100 nM of each primer, in 384 well plates (Sarstedt 72.1982.202) on a Roche LightCycler 480. Settings were: 95° C (5 min), 55 × [95° C (15 s), 56° C (15 s), 72° C (20 s)], melting curve [95° C (5 s), 65° C (1min), ramp to 97° C (0,11° C/min)], 40° C (30 s). Primers used in this study:

Gene	Fwd primer	Rev primer
18S	gtaacccgttgaaccccatt	ccatccaatcggtagtagcg
ACTB	aggcaccagggcgtgat	gcccacataggaatccttctgac
ANP32B	gcttacctacttggatgg	ctcatcttctccttcttcg
CTC338M12.4	ctacctgccttttggggaag	gaatggaagtggtgcctctg
EREG	gcacagctttagttcagac	actcatgtccaccagatag
IGFBP3	gctacaaagttgactacgag	gtcttccatttctctacgg
MAP6	ggc atg gac gga cat caa g	gcgtattcttctgcgatcaatga
OTOGL	gagtacgaaacttgtctgtg	tacagtctatgggttcctg
PAPPA	gggtatgtgaggagtttg	ctggtcttgatgagatactg
SGMS2	actctcaggcaaaagttc	gtcagtgtcagcgtaacc
TCF4	gtccactttccatcgtag	atactgtctgtcccgttg
TRIM41	ttctgccgagtttgtgtaacc	cctcctcctcccgatctaac
TRIM52	ggtgcaggagtaccaggaaataa	ataggccttgctgtgaatgct
TRIM52-AS1	agagcaaggactgtatgtgttc	ctggagtggcagaagtaagg

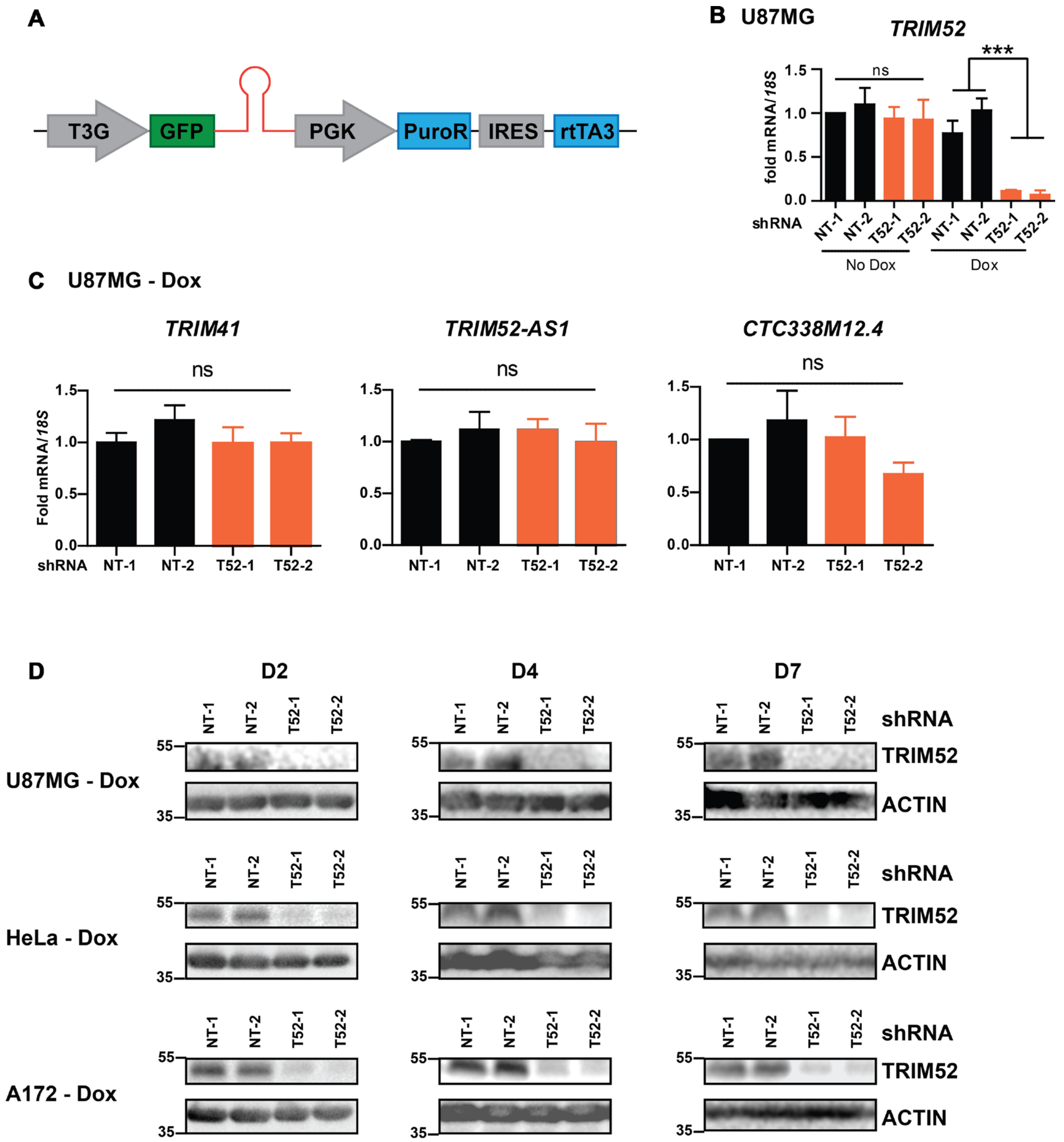

Supplementary Figure 1: *TRIM52*-specific shRNAs do not target *TRIM41* mRNA or lncRNAs expressed from *TRIM52* proximal loci. (**A**) Schematic of the used lentiviral dox-inducible shRNA vector. Dox-inducible promoter (T3G) drives expression of GFP and miRE based shRNA in the 3' UTR of GFP; phosphoglycerate kinase 1 (PGK) promoter drives expression of a puromycin resistance cassette (PuroR) and dox-controlled trans-activator 3 (rtTA3). (**B**) U87MG cells stably transduced with dox-inducible shRNA vectors were treated with dox for 4 days, and subsequently *TRIM52* mRNA expression was analyzed by RT-qPCR. Data are representative of at least three independent experiments. Represented are mean ± SD, $n = 3$; one way ANOVA followed by student's *t*-test was performed ($^{*}p > 0.05$; $^{**}p > 0.01$; $^{***}p > 0.001$). (**C**) U87MG cells stably transduced with dox-inducible shRNA vectors were treated with dox for 4 days. Expression of indicated mRNAs was analyzed by RT-qPCR relative to 18S rRNA. Data are representative of two independent experiments; mean ± SD, $n = 3$; student's *t*-test compared to NT-shRNAs was performed. (**D**) U87MG, HeLa, and A172 cells stably transduced with dox-inducible shRNA vectors were treated with dox for the indicated times, after which their TRIM52 protein levels were analyzed by WB.

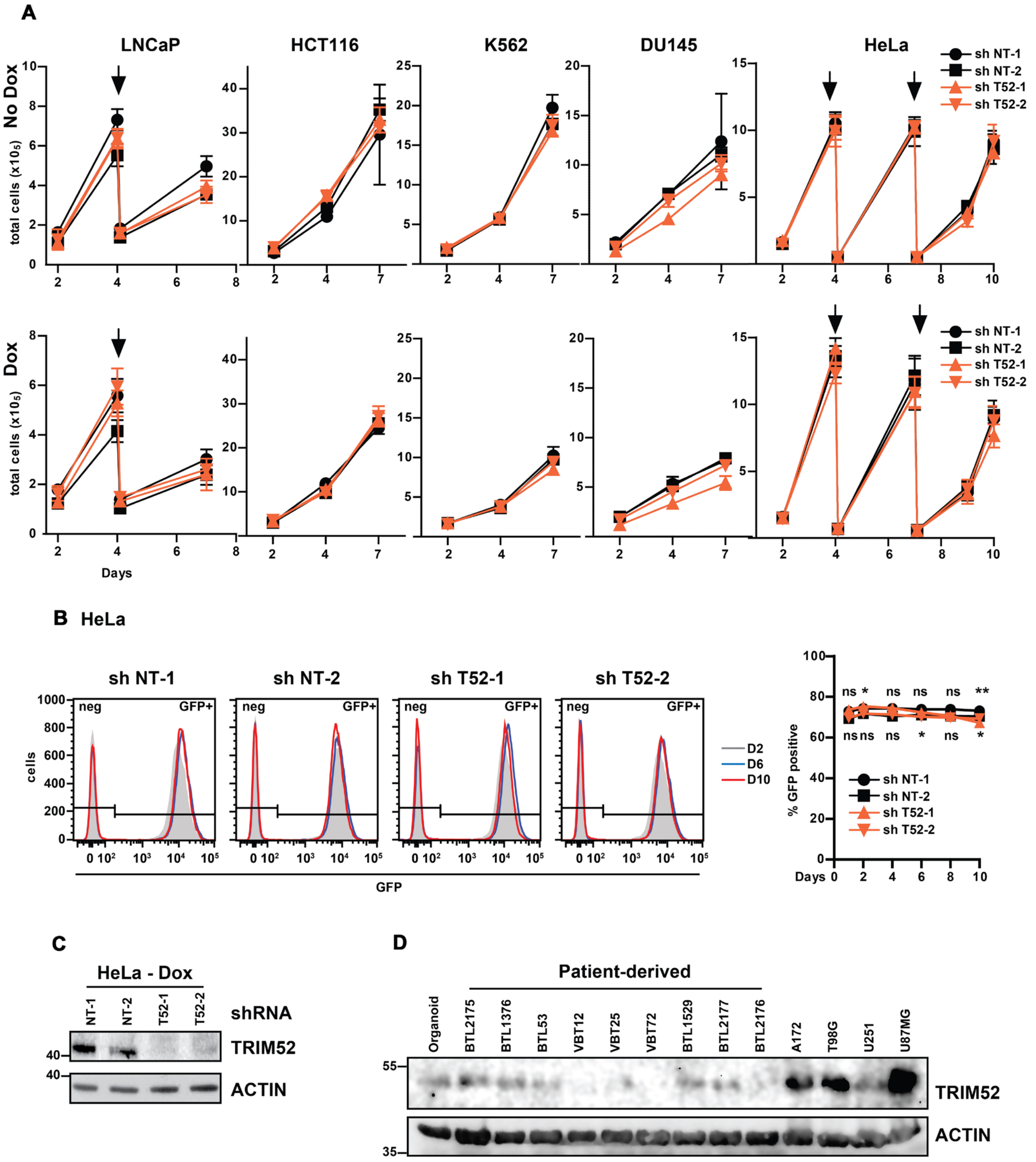

Supplementary Figure 2: *TRIM52* ablation does not affect fitness of various cancer cell lines. (**A**) LNCaP prostate carcinoma, HCT116 colon carcinoma, K562 chronic myeloid leukemia, DU145 prostate carcinoma, or HeLa cervical carcinoma cells stably transduced with dox-inducible shRNA vectors were seeded at a density of 1×10^5 cells/well. Cells were treated with dox and total cell numbers were counted 2, 4 and 7 days after seeding. HeLa cells reached confluency at day 4 and day 7, and were thus passaged at a fixed ratio (1:20, indicated by arrow) and counted additionally on day 9 and 10. LNCaP cells were split at a fixed ratio (1:4) on day 4. Data represent mean ± SD, $n = 3$ (**B**) HeLa cells stably transduced with dox-inducible shRNA vectors were mixed with WT cells (80% shRNA cell lines, 20% WT cells) and treated with dox. Cells were passaged every two days at a fixed ratio of 1:5 and GFP fluorescence intensity (left panel) and percentage GFP-positive cells (right panel) were measured by flow cytometry. Data represent mean ± SD, $n = 3$; student's *t*-test was performed on T52-1 or T52-2 shRNA cells compared to NT-shRNA cells; $^{*}p > 0.05$; $^{**}p > 0.01$.(**C**) TRIM52 Western blot analysis of dox-treated HeLa cells. (**D**) TRIM52 protein expression was analyzed by Western blot analysis in whole cell extracts from a human ES-derived cerebral organoid, and 9 patient-derived glioblastoma cell lines.

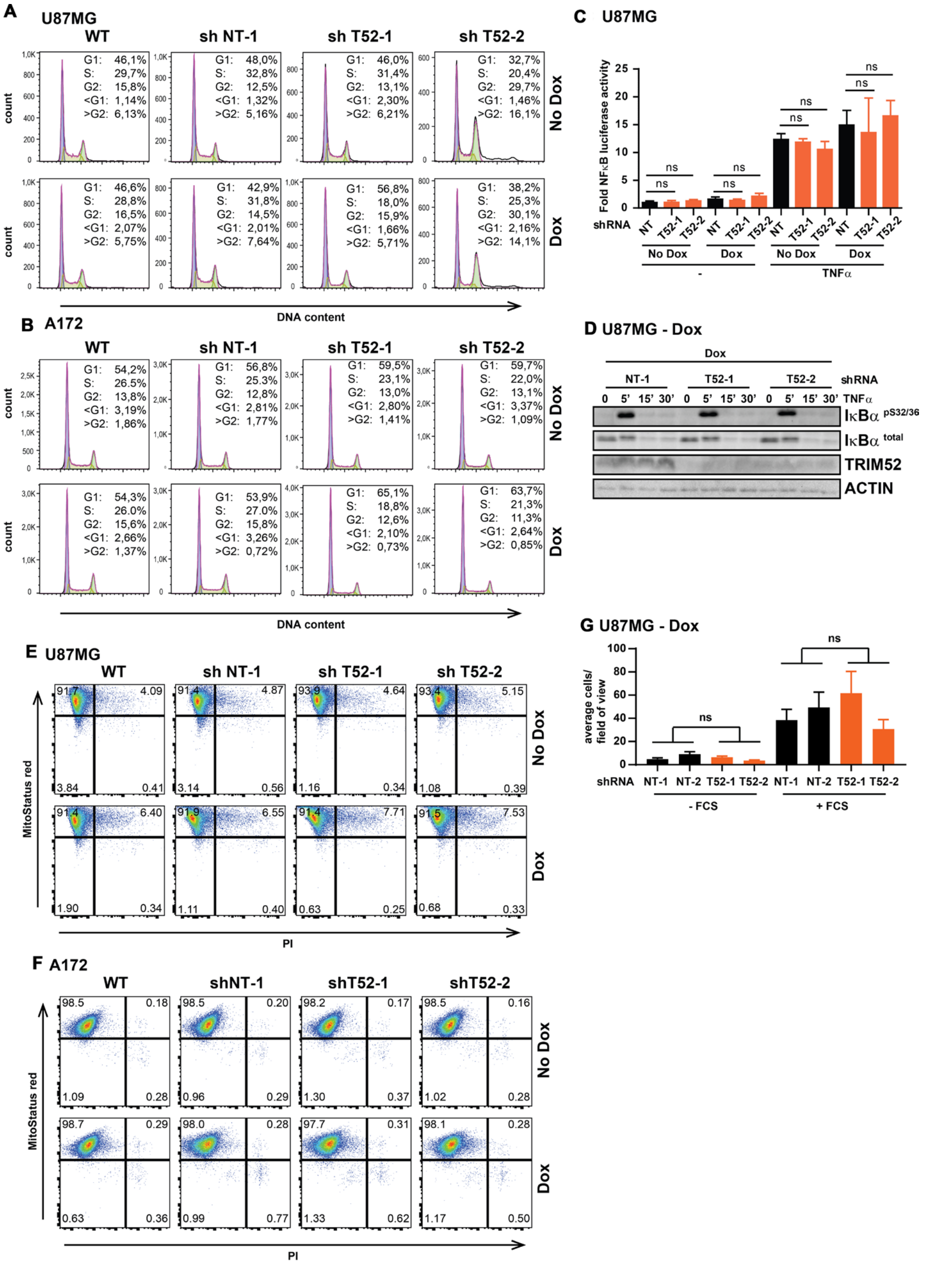

Supplementary Figure 3: TRIM52 regulates cell cycle, but not apoptosis or migration. (**A, B**) Representative FACS blots from (A) U87MG cells and (B) A172 cells corresponding to Figure 3A and 3B, respectively. (**C**) U87MG cells stably transduced with dox-inducible shRNA vectors were treated with dox for 4 days. Subsequently, cells were transfected with an NFκB-firefly luciferase and constitutively expressed renilla luciferase reporter plasmids. After 24 h, cells were stimulated with TNFα for another 24 h, after which dual-luciferase assays were performed. Data are representative of at least two independent experiments, $n = 3$, student's t-test was performed as indicated. (**D**) U87MG cells as in c. were treated with TNFα for the indicated time points and analyzed by WB for total IκBα and p- IκBα. Representative of at least 3 independent experiments. (**E, F**) Representative FACS plots of U87MG and A172 cells corresponding to Figure 3E and 3F, respectively. (**G**) U87MG cells stably transduced with dox-inducible shRNA vectors were treated with dox for 4 days. Cells were seeded without serum in porous tissue culture inserts. Bottom compartments either contained serum-free medium, or supplemented with 10% FCS as indicated. Migrated cells were counted ($n = 3$, student's t-test was performed as indicated: $^{**}p > 0.01$).

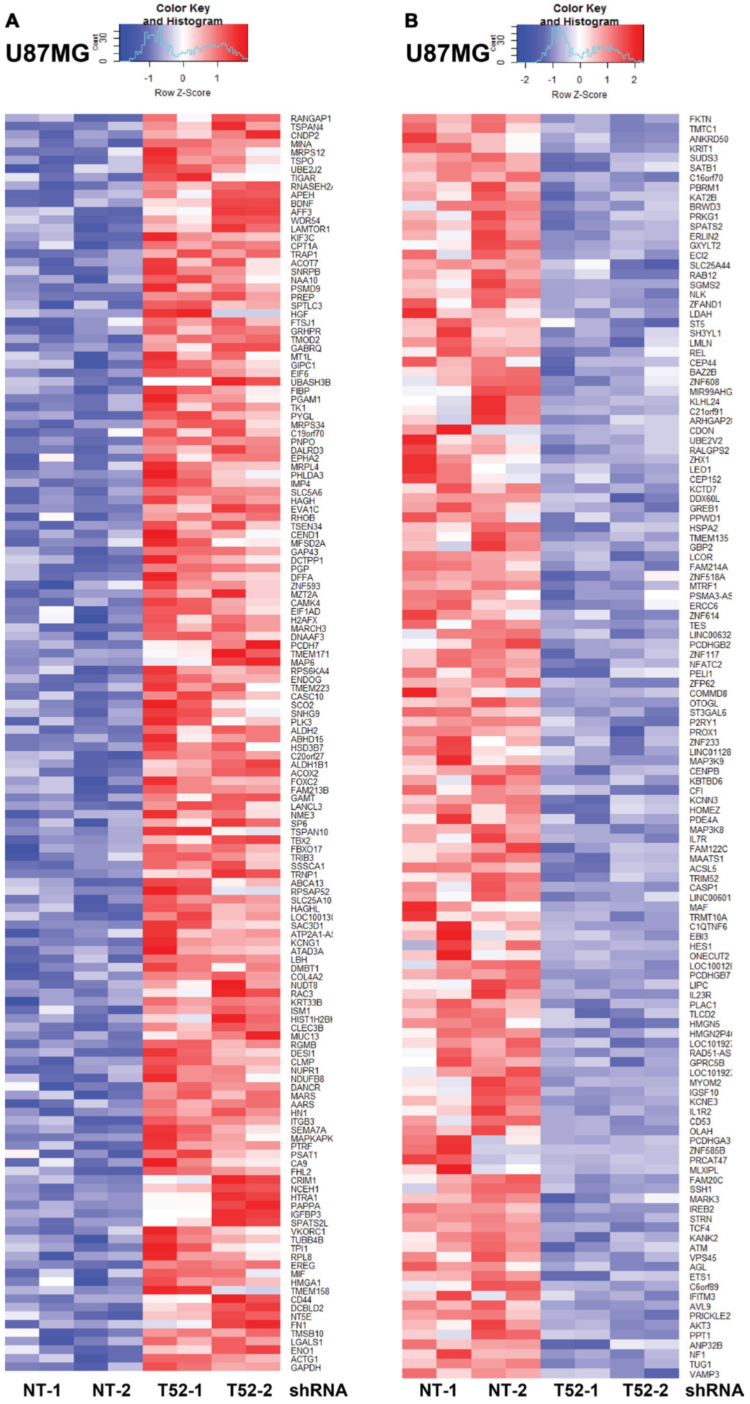

Supplementary Figure 4: Differentially expressed genes in *TRIM52* knockdown U87MG cells identified by mRNAseq. (**A, B**) U87MG cells stably transduced with dox-inducible shRNA vectors were treated with dox for 5 days, and differential expression was analyzed by mRNAseq. A cutoff of $p > 0.01$ was applied. All (**A**) up-regulated, or (**B**) down-regulated genes are indicated.

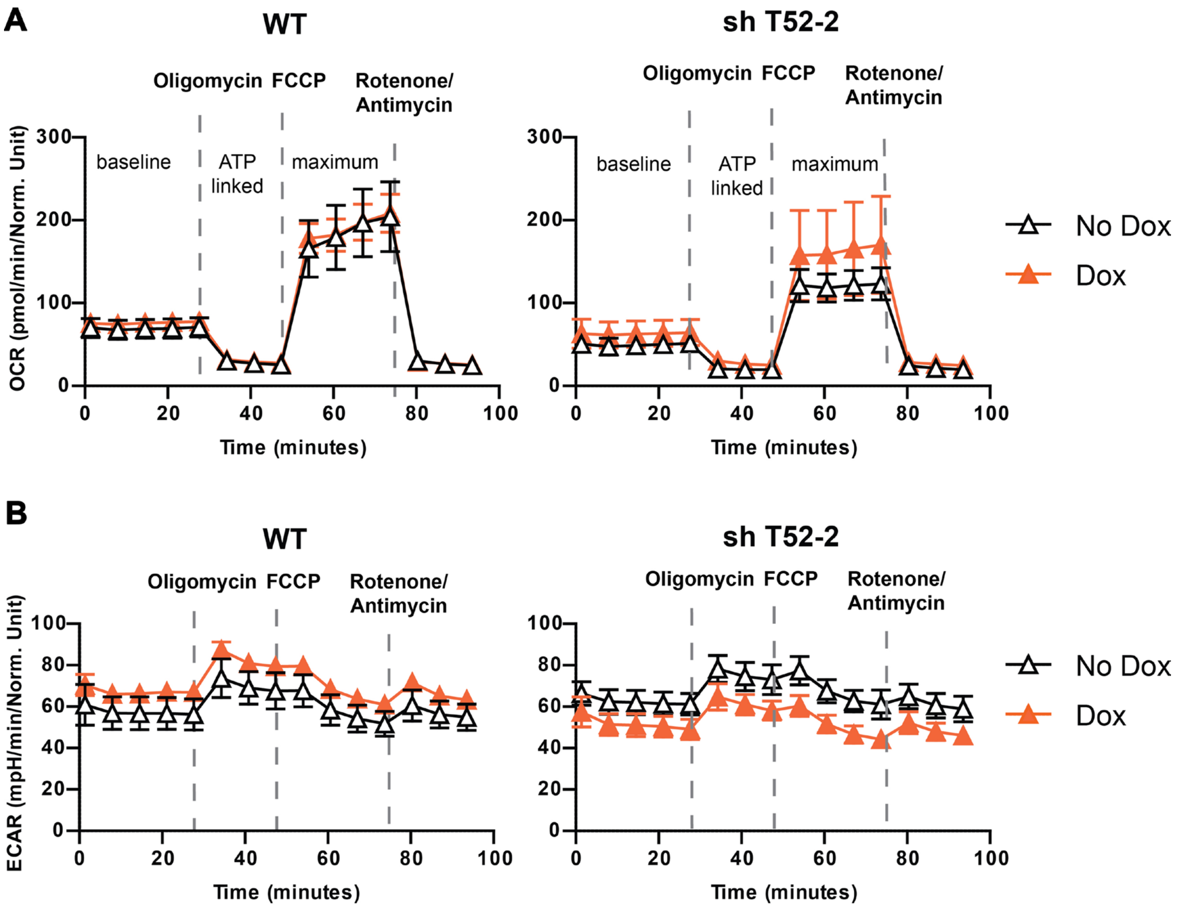

Supplementary Figure 5: *TRIM52* knockdown does not affect Oxygen Consumption Rate (OCR) and Extracellular Acidification Rate (ECAR). U87MG cells stably transduced with dox-inducible shRNA vectors were treated with dox for 4 days, and seeded into Seahorse bio-analyzer analysis plates. Measurements were carried out for up to 90 min. At indicated time points, oligomycin, FCCP or rotenone/antimycin A were injected into the wells. (**A**) Oxygen consumption rate (OCR) was measured and normalized to protein content. Fold basal (bottom left panel) and maximum (bottom right panel) OCR is displayed (n = 8–11). (**B**) Extracellular acidification rate (ECAR) was measured and normalized to protein content. Fold basal (bottom panel) ECAR is displayed (n = 8–11).

Supplementary Table 1: Mutational status of cell lines used in this study. See Supplementary_Table_1

Supplementary Table 2: Genes differentially regulated upon *TRIM52* knockdown in U87MG glioblastoma cells. See Supplementary_Table_2
