# Supplementary material for: Human tripartite motif protein 52 is required for cell context-dependent proliferation
Source: Oncotarget. 2018 Feb 5;9(17):13565–81. doi: 10.18632/oncotarget.24422 (PMC5862599; doi:10.18632/oncotarget.24422)
Supplement: Supplementary file 2 [file oncotarget-09-13565-s002.docx]

**Supplementary Table 1: mutational status of cell lines used in this study**

| Cell line | Cell type | TP53 | | | | | PTEN | | | | |
| --- | --- | --- | --- | --- | --- | --- | --- | --- | --- | --- | --- |
|  |  | **status** | **DNA** | **Protein** | **Zygosity** | **Mutation Type** | **status** | **DNA** | **Protein** | **Zygosity** | **Mutation type** |
| U87MG [1–3] | Glioblastoma | WT |  |  |  |  | mut | c.209+1G>T | p.? | Hom | mutated splice site, not expressed |
| U251 [1,4–7] | Glioblastoma | mut | c.818G>A | p.R273H | Hom | Oncogenic gain of function | mut | c.723_724insTT | p.E242fsX15 | Hom | ? |
| T98G [1,8–12] | Glioblastoma | mut | c.711G>A | p.M237I | Hom | Reduced stability and transactivation | mut | c.125T>G | p.L42R | Hom | loss of function |
| A172 [1,13,14] | Glioblastoma | WT |  |  |  |  | mut | c.165_1212del1048 | p.R55fs*1 | Hom | loss of function/truncation |
| LNCaP [1,15–17] | Prostate carcinoma | WT |  |  |  |  | mut | c.17_18delAA | p.K6fs*4 | Hom | deletion/frameshift |
| DU145 [1,16,18,19] | Prostate carcinoma | mut | c.820G>T  c.668C>T | p.V274F  p.P223L | Het | Non functional  Temperature sensitive | WT |  |  |  |  |
| HCT116 [7] | Colorectal carcinoma | WT |  |  |  |  | WT |  |  |  |  |
| HeLa [20–22] | Cervix carcinoma | WT* |  |  |  | Deactivated and degraded | WT |  |  |  |  |
| K562 [1,7,23] | Chronic myeloid leukemia | mut | c.406_407insC | p.Q136fs*13 | Hom | RNA and protein undetectable | WT |  |  |  |  |

**References:**

1. ATTC Cell lines by gene mutation. Available from https://www.atcc.org/~/media/PDFs/Culture%20Guides/Cell_Lines_by_Gene_Mutation.ashx

2. Clark MJ, Homer N, O’Connor BD, Chen Z, Eskin A, Lee H, Merriman B, Nelson SF. U87MG Decoded: The Genomic Sequence of a Cytogenetically Aberrant Human Cancer Cell Line. PLOS Genet. 2010; 6: e1000832. doi: 10.1371/journal.pgen.1000832.

3. Sawada T, Hamano N, Satoh H, Okada T, Takeda Y, Mabuchi H. Mutation analysis of the PTEN / MMAC1 gene in Japanese patients with Cowden disease. Jpn J Cancer Res Gann. 2000; 91: 700–5.

4. The TP53 website. Available from http://p53.free.fr/Database/Cancer_cell_lines/p53_cell_lines.html

5. Gomez-Manzano C, Fueyo J, Kyritsis AP, McDonnell TJ, Steck PA, Levin VA, Yung WK. Characterization of p53 and p21 functional interactions in glioma cells en route to apoptosis. J Natl Cancer Inst. 1997; 89: 1036–44.

6. Sesen J, Dahan P, Scotland SJ, Saland E, Dang VT, Lemarié A, Tyler BM, Brem H, Toulas C, Cohen-Jonathan Moyal E, Sarry JE, Skuli N. Metformin inhibits growth of human glioblastoma cells and enhances therapeutic response. PloS One. 2015; 10: e0123721. doi: 10.1371/journal.pone.0123721.

7. Ikediobi ON, Davies H, Bignell G, Edkins S, Stevens C, O’Meara S, Santarius T, Avis T, Barthorpe S, Brackenbury L, Buck G, Butler A, Clements J, et al. Mutation analysis of 24 known cancer genes in the NCI-60 cell line set. Mol Cancer Ther. 2006; 5: 2606–12. doi: 10.1158/1535-7163.MCT-06-0433.

8. Van Meir EG, Kikuchi T, Tada M, Li H, Diserens AC, Wojcik BE, Huang HJ, Friedmann T, de Tribolet N, Cavenee WK. Analysis of the p53 gene and its expression in human glioblastoma cells. Cancer Res. 1994; 54: 649–52.

9. Hill VK, Kim JS, James CD, Waldman T. Correction of PTEN mutations in glioblastoma cell lines via AAV-mediated gene editing. PloS One. 2017; 12: e0176683. doi: 10.1371/journal.pone.0176683.

10. Steck PA, Pershouse MA, Jasser SA, Yung WK, Lin H, Ligon AH, Langford LA, Baumgard ML, Hattier T, Davis T, Frye C, Hu R, Swedlund B, et al. Identification of a candidate tumour suppressor gene, MMAC1, at chromosome 10q23.3 that is mutated in multiple advanced cancers. Nat Genet. 1997; 15: 356–62. doi: 10.1038/ng0497-356.

11. Jordan JJ, Inga A, Conway K, Edmiston S, Carey LA, Wu L, Resnick MA. Altered-function p53 missense mutations identified in breast cancers can have subtle effects on transactivation. Mol Cancer Res. 2010; 8: 701–16. doi: 10.1158/1541-7786.MCR-09-0442.

12. Nguyen HN, Yang JM, Rahdar M, Keniry M, Swaney KF, Parsons R, Park BH, Sesaki H, Devreotes PN, Iijima M. A new class of cancer-associated PTEN mutations defined by membrane translocation defects. Oncogene. 2015; 34: 3737–43. doi: 10.1038/onc.2014.293.

13. Sauvageot CM, Weatherbee JL, Kesari S, Winters SE, Barnes J, Dellagatta J, Ramakrishna NR, Stiles CD, Kung AL, Kieran MW, Wen PY. Efficacy of the HSP90 inhibitor 17-AAG in human glioma cell lines and tumorigenic glioma stem cells. Neuro Oncol. 2009; 11: 109–21. doi: 10.1215/15228517-2008-060.

14. Mendes-Pereira AM, Martin SA, Brough R, McCarthy A, Taylor JR, Kim JS, Waldman T, Lord CJ, Ashworth A. Synthetic lethal targeting of PTEN mutant cells with PARP inhibitors. EMBO Mol Med. 2009; 1: 315–22. doi: 10.1002/emmm.200900041.

15. Chappell WH, Lehmann BD, Terrian DM, Abrams SL, Steelman LS, McCubrey JA. p53 expression controls prostate cancer sensitivity to chemotherapy and the MDM2 inhibitor Nutlin-3. Cell Cycle Georget Tex. 2012; 11: 4579–88. doi: 10.4161/cc.22852.

16. Lotan TL, Gurel B, Sutcliffe S, Esopi D, Liu W, Xu J, Hicks JL, Park BH, Humphreys E, Partin AW, Han M, Netto GJ, Isaacs WB, et al. PTEN protein loss by immunostaining: analytic validation and prognostic indicator for a high risk surgical cohort of prostate cancer patients. Clin Cancer Res. 2011; 17: 6563–73. doi: 10.1158/1078-0432.CCR-11-1244.

17. Catalogue of somatic mutations in cancer (COSMIC). Available from http://cancer.sanger.ac.uk/cosmic/mutation/overview?id=4929

18. International agency for research on cancer (IARC) TP53 database. Available from http://p53.iarc.fr/TP53GeneVariations.aspx?mutant=V274F

19. Bajgelman MC, Strauss BE. The DU145 human prostate carcinoma cell line harbors a temperature-sensitive allele of p53. The Prostate. 2006; 66: 1455–62. doi: 10.1002/pros.20462.

20. Hietanen S, Lain S, Krausz E, Blattner C, Lane DP. Activation of p53 in cervical carcinoma cells by small molecules. Proc Natl Acad Sci U S A. 2000; 97: 8501–6.

21. Kralj M, Husnjak K, Körbler T, Pavelić J. Endogenous p21WAF1/CIP1 status predicts the response of human tumor cells to wild-type p53 and p21WAF1/CIP1 overexpression. Cancer Gene Ther. 2003; 10: 457–67. doi: 10.1038/sj.cgt.7700588.

22. Ajay AK, Meena AS, Bhat MK. Human papillomavirus 18 E6 inhibits phosphorylation of p53 expressed in HeLa cells. Cell Biosci. 2012; 2: 2. doi: 10.1186/2045-3701-2-2.

23. Forbes S, Clements J, Dawson E, Bamford S, Webb T, Dogan A, Flanagan A, Teague J, Wooster R, Futreal PA, Stratton MR. COSMIC 2005. Br J Cancer. 2006; 94: 318.
